# Metal–organic frameworks of *p*-hydroxybenzoic acid: synthesis, structure and ring opening polymerization capability†

**DOI:** 10.1039/d4ra06361a

**Published:** 2024-11-27

**Authors:** Yi Gong, Simon J. Sharp, Mark R. J. Elsegood, Carl Redshaw

**Affiliations:** a Chemistry, School of Natural Sciences, University of Hull Hull HU6 7RX UK C.Redshaw@hull.ac.uk; b Chemistry Department, Loughborough University Loughborough LE11 3TU UK m.r.j.elsegood@lboro.ac.cuk

## Abstract

Two new, isostructural, metal–organic frameworks {[Co(O_2_CC_6_H_4_O)(DMF)]_2_}_*n*_ and {[Mn(O_2_CC_6_H_4_O)(DMF)]_2_}_*n*_ have been synthesised and structurally characterized. Use of *p*-hydroxybenzoic acid resulted in a three-dimensional MOF featuring a linker with a carboxylic group and a *para*-hydroxyl group. Ring opening polymerization of ε-caprolactone and δ-valerolactone has been performed using these MOFs as catalysts, and results compared with the known zinc MOF Zn(O_2_CC_6_H_4_O). The resulting products are predominantly cyclic polymers. The manganese and zinc MOFs exhibit significant recyclability during ring opening polymerization.

## Abstract 2

Two new, isostructural, MOFs {[Co(O_2_CC_6_H_4_O)(DMF)]_2_}_*n*_ and {[Mn(O_2_CC_6_H_4_O)(DMF)]_2_}_*n*_ have been synthesised and structurally characterized. Their use as catalysts in the ROP of ε-caprolactone or δ-valerolactone produces mostly cyclic products.

## Graphical abstract

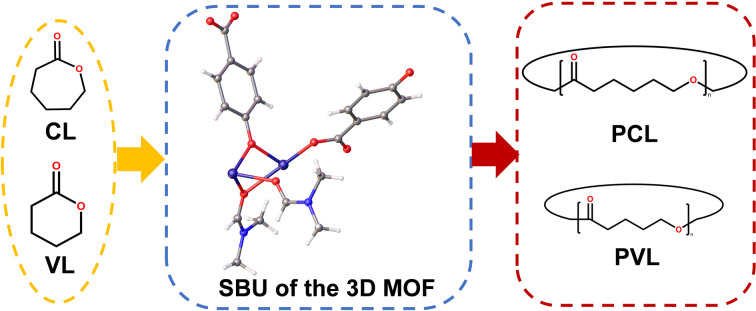

## Introduction

Since the work by Yaghi *et al.* detailing the metal–organic framework MOF-5 and subsequent publications detailing the ability of this MOF as a robust heterogeneous catalyst, as well as various other applications including as a sensor and gas storage material,^1–3^ there has been a huge amount of research into the construction of MOFs utilising the linker 1,4-benzenedicarboxylate (BDC). Whilst many MOF systems have been constructed using this BDC linker in conjunction with a variety of metals, the use of MOFs in catalysis still remains an emerging area.^4–16^ Work by Guo *et al.* found that the catalytic selectivity can be regulated by the topology of the chiral metal–organic frameworks (CMOFs), with different topologies leading to variations in the spatial location and orientation of the active and chiral sites.^17^ Such work inspired us to explore unusual or novel topologies and secondary building units (SBUs) of MOFs, and to evaluate their catalytic performance.

Approaches to improve the potential wider use of MOFs as catalysts in ring opening polymerization (ROP), include introducing more diverse transition metals^18,19^ and using other oxygen-containing functional groups.^20,21^ The benefits of the tuneable framework, together with the reasonable stability, are indeed leading to studies of heterogenous MOF catalysts as efficient systems for the ROP of cyclic esters.^22^

**Scheme 1:**
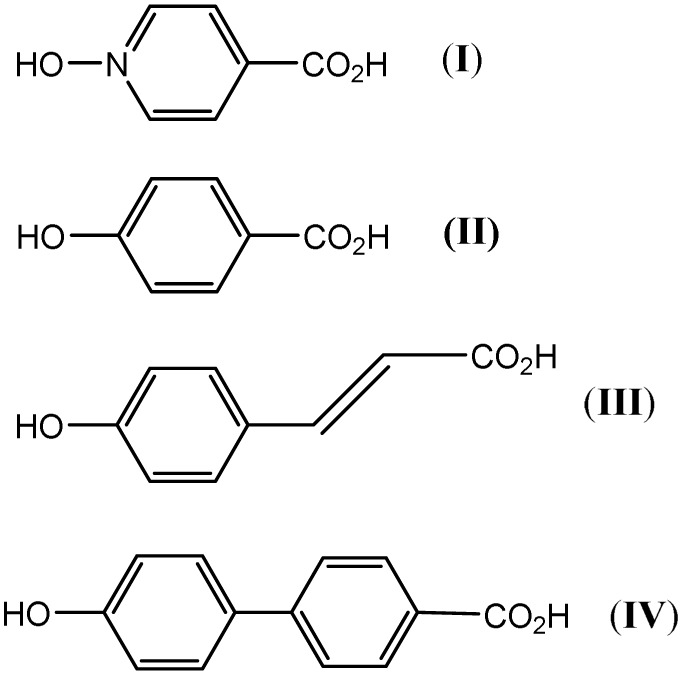
Linkers employed by Abrahams, Robson *et al.*^23^

One ligand set that attracted our attention involved the combination of phenolic and carboxylic acid end groups. Abrahams, Robson, *et al.* reported a series of coordination polymers derived from the phenolic carboxylic acids I to IV (Scheme 1), and employed the resulting polymers for gas (CH_4_, H_2_, or CO_2_) storage.^23^ Despite the similarity of this type of ligand set to the very popular BDC type linker, it has received far less attention.

With this in mind, we have initiated research into MOFs based on the potential organic linker *p*-hydroxybenzoic acid (II). It is hoped that the use of a ligand with a single carboxylic function and *para*-hydroxyl group will impart different methods of metal binding, promoting the formation of new SBUs.

In this paper, the preparation of {[Co(O_2_CC_6_H_4_O)(DMF)]_2_}_*n*_ (1) and {[Mn(O_2_CC_6_H_4_O)(DMF)]_2_}_*n*_ (2) were achieved *via* the solvothermal reaction of *p*-hydroxybenzoic acid with the hydrated metal nitrates of cobalt and manganese in DMF (*N*,*N*′-dimethylformamide). Slow cooling of the reaction vessels led to the formation of crystals of suitable quality for X-ray diffraction. Additionally, the zinc MOF (Zn-hba, 3),^23^ which utilizes the same linker but is synthesized *via* a different method (involving boiling methanol), was prepared in order to compare its catalytic performance with that of MOFs 1 and 2.

## Experimental

### General

Dichloromethane, acetic acid and methanol were purchased from Fisher Chemical. Tetrahydrofuran extra dry was purchased from Thermo Scientific. *N*,*N*′-Dimethylformamide, *p*-hydroxybenzoic acid, cobalt(ii) acetate tetrahydrate and ε-caprolactone were purchased from Alfa Aesar. Manganese(ii) acetate tetrahydrate and δ-valerolactone were purchased from Sigma-Aldrich. All reagents were used without further purification. An electric balance (Sartorius Analytical A200s) was utilized to weigh the chemicals. The hydrothermal reactions were conducted in sealed glass tubes. Catalysts were dried in a vacuum oven at 50 °C overnight before the ROP reactions. Infra-red (IR) results were collected from an iD7 Transmission Diamond ATR, Nicolet iS5 FTIR spectrometer. Elemental analyses were performed at London Metropolitan University. MALDI-TOF mass spectra were obtained on a Bruker Maxis Impact HD Mass spectrometer in ESI positive mode. Thermogravimetric analysis (TGA) results were recorded on a PerkinElmer TGA 4000 thermogravimetric analyser under ambient atmosphere from 30 to 757 °C. Powder X-ray diffraction (PXRD) patterns were acquired using a PANAlytical Empyrean Series 2 powder diffractometer with a copper X-ray tube. ^1^H NMR spectra were recorded on a JEOL ECZ 400S spectrometer operating at 400.2 MHz. Gel Permeation Chromatography (GPC) analyses were performed using a SHIMADZU liquid chromatography (LC-6A), a Viscotek VE 3580 RI detector, a Viscotek VE 5111 injector valve bracket and a Viscotek 270 dual detector. The molecular weights and polydispersity index were determined by analyzing the experimental traces with OmniSEC 5.12 software. The recycled catalyst was separated from polymers by dissolving the resulting product in CH_2_Cl_2_, followed by use of a centrifuge (Heraeus Megafuge 8, Thermo Scientific).

#### Synthesis of 1

Co(OAc)_2_·4H_2_O (0.250 g, 1.0 mmol) and *p*-hydroxybenzoic acid (0.14 g, 1.0 mmol) were dissolved in DMF (12 mL) and placed into a sealed flask. The flask was heated to 140 °C for 24 h. Slow cooling of the reaction flask yielded small purple prisms (80% yield). Elemental analysis cal. for {[Co(O_2_CC_6_H_4_O)(DMF)]_2_}_*n*_: C 44.79, H 4.14, N 5.22; found: C 44.56, H 3.97, N 4.52.

#### Synthesis of 2

Mn(OAc)_2_·4H_2_O (0.245 g, 1.0 mmol) and *p*-hydroxybenzoic acid (0.14 g, 1.0 mmol) were dissolved in DMF (12 mL) and placed into a sealed flask. The flask was heated to 140 °C for 24 h. Slow cooling of the reaction flask yielded small brown prisms (62% yield). Elemental analysis cal. for {[Mn(O_2_CC_6_H_4_O)(DMF)]_2_}_*n*_: C 45.47, H 4.20, N 5.30; found: C 44.53, H 3.58, N 4.94.

#### Synthesis of 3

The synthesis of Zn(O_2_CC_6_H_4_O) followed the literature method.^23^

### Procedure for the ROP of ε-caprolactone or δ-valerolactone

The ROP of ε-CL and δ-VL were conducted in the melt phase. The catalyst (pre-dried *in vacuo* for 12 h) was weighed (5.36 mg of 1, 5.28 mg of 2, 4.03 mg of 3) and the monomer (0.55 mL of ε-CL, 0.45 mL of δ-VL) was added into a test tube, and the reaction mixture was subsequently placed in a preheated sand bath at 130 °C. The reactions under nitrogen were conducted in a Schlenk flask connected to a Schlenk line. The reaction was then quenched using 0.2 mL of glacial acetic acid and 10 mL of cold methanol. The reaction conversion was monitored using ^1^H NMR spectroscopy. The resulting polymer was collected after solvent vaporization and dried in a fume cupboard. The molecular weights (*M*_n_) and polydispersity indices (*Đ*) of the polymer products were determined using gel permeation chromatography (GPC) in extra dry THF.

### Kinetics studies

Kinetic experiments were carried out according to the previously described polymerization protocol. At appropriate intervals, approximately 0.05 mL of the reaction mixture was sampled, quenched with 1 mL of cold deuterated chloroform, and analyzed using ^1^H NMR spectroscopy (in CDCl_3_).

## Results and discussion

### Structures of 1 and 2

**Fig. 1:**
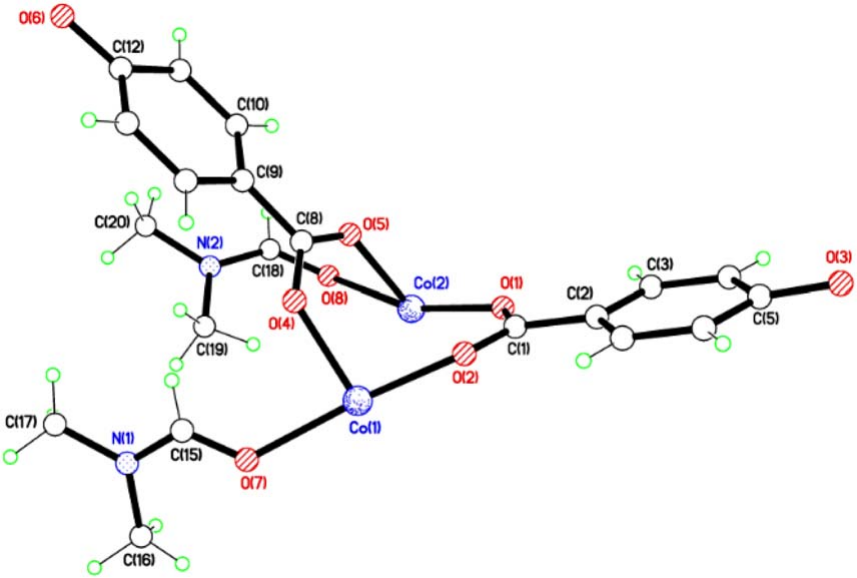
Structure of the asymmetric unit of 1; That of 2 is virtually identical. Selected bond lengths (Å) and angles (°) for 1: Co(1)–O(2) 2.028(3), Co(1)–O(4) 2.025(6), Co(1)–O(7) 2.152(4), Co(1)–O(3A) 2.077(3), Co(1)–O(6A) 2.042(4), Co(1)–O(8A) 2.313(5), Co(2)–O(1) 1.977(4), Co(2)–O(5) 2.025(5), Co(2)–O(8) 2.367(5), Co(2)–O(6A) 2.019(4); Co(1A)–O(8)–Co(2) 84.84(16), Co(1A)–O(3)–Co(2A) 105.07(15), O(3A)–Co(1)–O(4) 97.6(3), O(2)–Co(1)–O(4) 98.0(3), O(8)–Co(2)–O(1) 160.39(17), O(1)–Co(2)–O(6A) 98.41(15). For 2: Mn(1)–O(2) 2.098(3), Mn(1)–O(4) 2.114(3), Mn(1)–O(7) 2.285(3), Mn(2)–O(1) 2.090(3), Mn(2)–O(5) 2.113(3), Mn(2)–O(8) 2.363(3); O(2)–Mn(1)–O(4) 99.68(13), O(2)–Mn(1)–O(7) 165.79(12), O(1)–Mn(2)–O(5) 100.66(14), O(1)–Mn(2)–O(8) 166.23(13), O(5)–Mn(2)–O(8) 80.80(13).

**Fig. 2:**
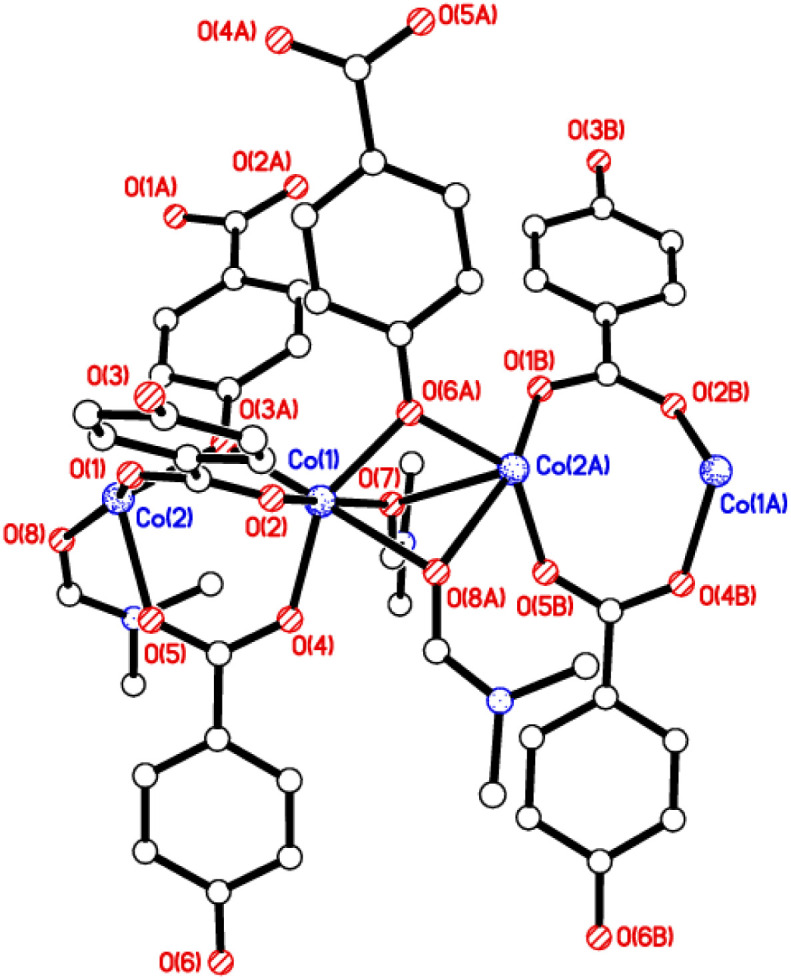
View of the chains aligned parallel to the *b* axis in 1 and 2.

**Fig. 3:**
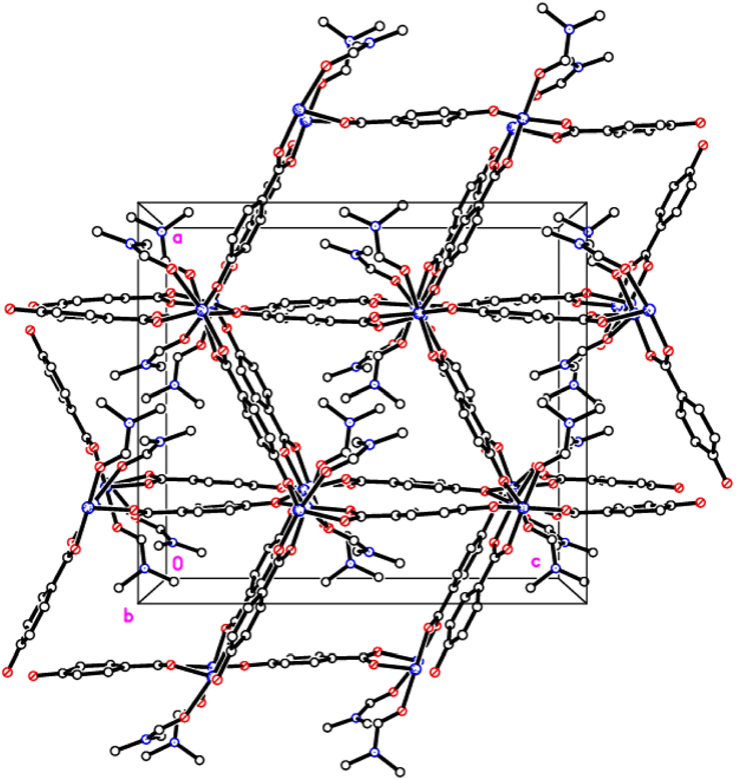
View down the channels (*b* axis) in 1 and 2.

The crystal structures of {[M(O_2_CC_6_H_4_O)(DMF)]_2_}_*n*_ (M = Co, 1; M = Mn, 2) are almost isostructural and reveal a 3D MOF structure with DMFs pointing into the diamond-shaped channels aligned parallel to *b* (Fig. 1–3 and with further details in the ESI).† Each pair of M^2+^ ions is triply bridged, but in two different ways. In the first the bridging is *via* two carboxylate groups and a phenolate oxygen, O(3), with M⋯M separations of 3.260 and 3.380 Å for M = Co and Mn, respectively. In the second it is *via* single oxygen bridges of the two unique DMF oxygens and the other unique phenolate oxygen, O(6), with shorter M⋯M separations of 3.157 and 3.226 Å for M = Co and Mn, respectively. Aromatic ligands including O(6) form flat layers parallel to the *b*/*c* plane, while those involving O(3) form the links between these planes on a diagonal. There is no clear path through the lattice parallel to *c* due to aromatic rings blocking the way. The metal centres are either 6-coordinate, as in Co(1)/Mn(1), bound by two phenolic oxygens, two carboxylato oxygens and two DMF molecules (one terminal, one bridging), or 5-coordinate, as for Co(2)/Mn(2), for which there is no terminal DMF ligand.

**Fig. 4:**
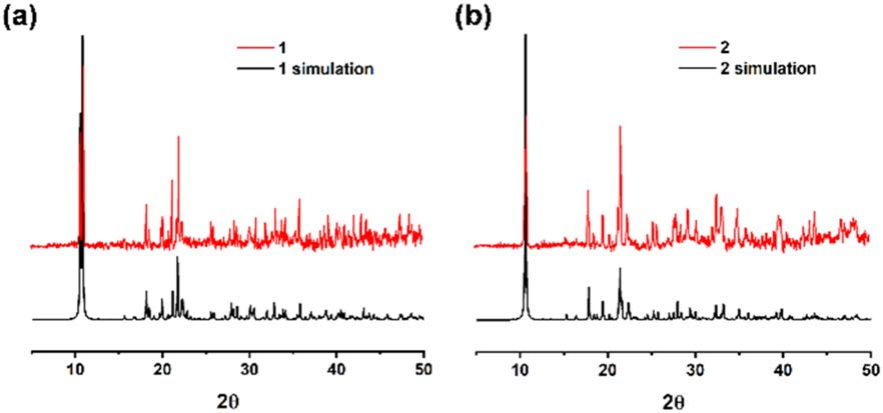
X-ray powder diffraction patterns of (a) 1, (b) 2 and their simulations.

X-ray powder diffraction patterns of 1 and 2 confirmed the high purity, crystallinity, and similarity of these two products (Fig. 4). MOF 3 was synthesized according to the literature and also shows good agreement with both the experimental and simulated data (Fig. S1, ESI†). The peaks from the same functional groups from hydroxybenzoic acid were observed in the infrared spectra of 1–3 (Fig. S2, ESI†). The peaks at 1452–1685 cm^−1^ correspond to the stretching vibration of the acidic C=O bonds. The peaks at 1057–1452 cm^−1^ are attributed to the stretching vibration of the aromatic ester C–O bonds. Additionally, the peaks at 540–902 cm^−1^ are characteristic of the aromatic C–H bonds.

### Ring opening polymerization

**Fig. 5:**
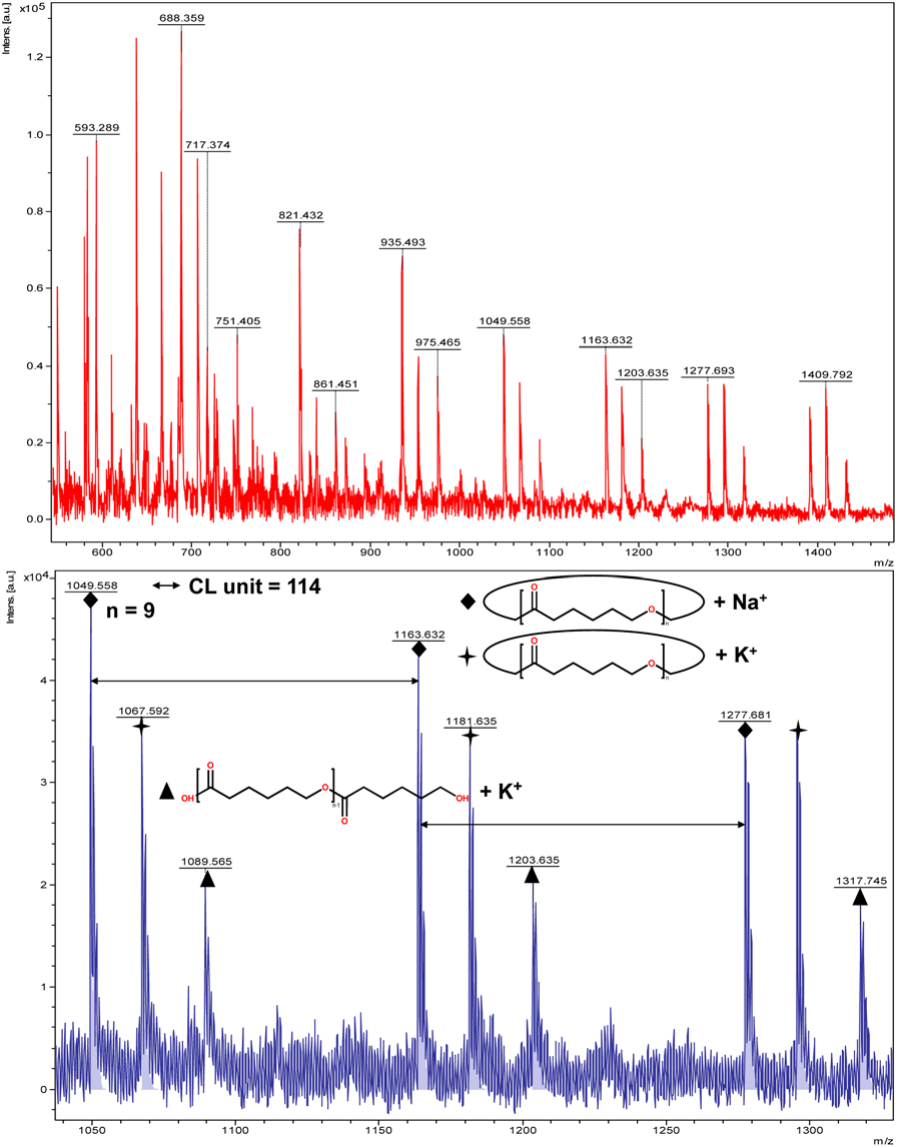
MALDI-TOF mass spectrum of PCL (Table 1, entry 5).

**Fig. 6:**
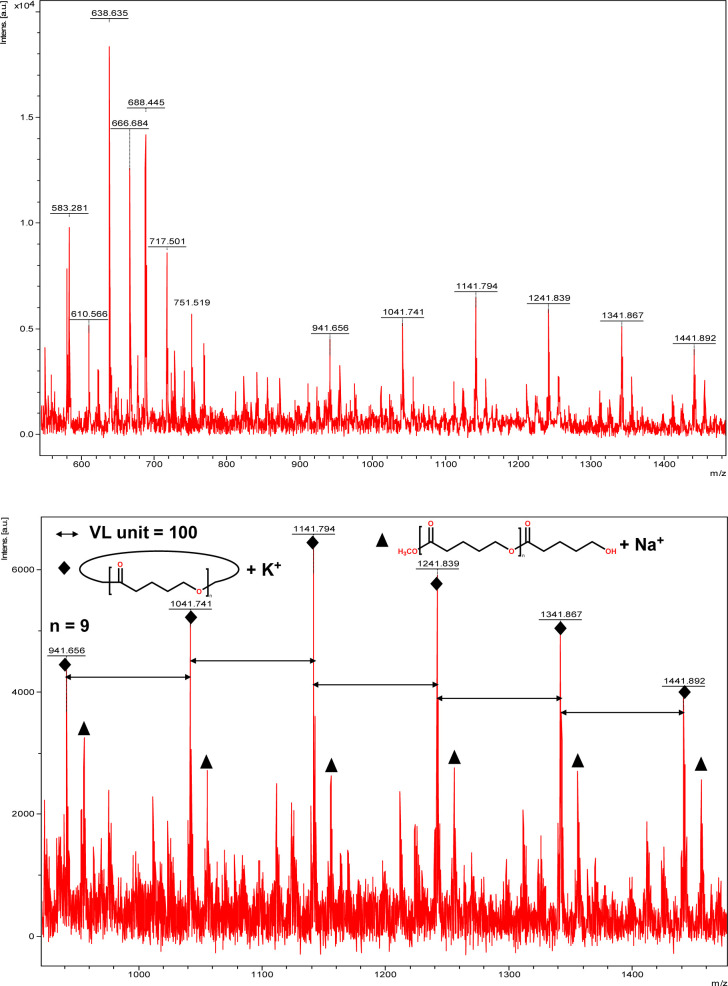
MALDI-TOF mass spectrum of PVL (Table 1, entry 2).

The catalytic performance of 1–3 in the ring opening polymerization (ROP) of ε-CL or δ-VL was investigated. All reactions were conducted in a solvent-free environment, *i.e.* as melts, as all catalysts demonstrated no activity in the presence of a solvent (toluene). Table 1 gives the results of the ROP of ε-CL or δ-VL initiated by 1–3 under air at 130 °C. At a [Cat] : [monomer] ratio of 1 : 500, nearly 100% conversion (*e.g.* conversion = 100%, Table 1, entry 2) was achieved in all cases, except for the ROP of ε-CL initiated by 2, which did not reach complete conversion but afforded a narrower polydispersity (*Đ* = 1.44, Table 1, entry 3). Furthermore, at different ratios of 1 : 250 and 1 : 750, the ROP of ε-CL or δ-VL initiated by 1 exhibited good catalytic performances. The ROP reactions performed under nitrogen using a [Cat] : [monomer] ratio of 1 : 500 are recorded in Table 2. Under the same conditions (130 °C for 24 h), all cases revealed lower conversion compared with the ROP reactions conducted under air. End group analysis of the resulting PCL from Table 1, entries 1 and 5, indicates that the products are cyclic PCL (Fig. S7 and S11, ESI†). For the resulting PCL or PVL from Table 1, entries 2, 4, and 6, the products consist predominantly of cyclic polymers with a small amount of linear polymers (Fig. S8, S10 and S12, ESI†). Corresponding MALDI-TOF spectra are presented in Fig. 5 and S13–S17,† indicating the dominant cyclic products. For example, three types of peaks were observed in the MALDI-TOF spectrum of PCL obtained using 3 (run 5, Table 1 and Fig. 5). The peak at *m*/*z* 1049.558 is attributed to cyclic PCL with Na^+^ (*m*/*z*: 114.1 × *n* + 23.0, *n* = 9), whilst the peak at *m*/*z* 1067.592 is assigned to cyclic PCL with K^+^ (*m*/*z*: 114.1 × *n* + 39.1, *n* = 9). In addition, small amounts of linear polymers obtained *via* quenching the products with methanol were observed. The signal peak at *m*/*z* 1089.565 corresponds to linear PCL HO(CL)_*n*_H with K^+^ (*m*/*z*: 18.0 + 114.1 × *n* + 39.1, *n* = 9). Two types of peaks were observed in the MALDI-TOF spectrum of PVL obtained using 1 (run 2, Table 1 and Fig. 6). The peak at *m*/*z* 941.656 represents cyclic PVL with K^+^ (*m*/*z*: 100.1 × *n* + 39.1, *n* = 9), whilst the peak at *m*/*z* 955.703 is assigned to linear PVL H_3_CO(CL)_*n*_H obtained *via* quenching the products with methanol and glacial acetic acid with Na^+^ (*m*/*z*: 100.1 × *n* + 32.0 + 23.0, *n* = 9). For the ROP conducted under nitrogen in Table 2, it is observed that the conversion is generally lower than that conducted under air. This is most likely due to the hydrophilic nature of the monomer as reported by Repo *et al.*, and under air, the water present can be a co-initiator in the ROP.^24^ Interestingly, the ROP conversion with δ-VL is generally higher than with ε-CL, which is inconsistent with the thermodynamic parameters for these lactones.^25^ However, we have noted this trend in other systems, for example the use of dianilines as catalysts.^26^ In the ROP of δ-VL initiated by 2 and 3, the ROP of ε-CL initiated by 3 under nitrogen atmosphere, linear polymer is the main product and small amount of cyclic polymer formed, which are confirmed by ^1^H NMR (Fig. S17–S20†) and MALDI-TOF spectra (Fig. S21 and S23†). However, the MALDI-TOF results of the ROP of δ-VL initiated by 1 under nitrogen atmosphere indicates that cyclic PVL is the main product (Fig. S22†).

**Table 1:** ROP of ε-CL or δ-VL initiated by 1–3 under air^a^

Entry	Catalyst	[Cat] : [monomer]^b^	Time (h)	Conv.^c^ (%)	*M*_n_ d (Da)	*Đ* e	Yield^f^ (%)
1	1	CL, 1 : 500	24	98	11 790	3.04	92
2	1	VL, 1 : 500	24	100	11 440	3.28	66
3	2	CL, 1 : 500	24	26	3820	1.44	100
4	2	VL, 1 : 500	24	99	12 670	2.29	71
5	3	CL, 1 : 500	24	100	5520	2.10	99
6	3	VL, 1 : 500	24	100	3440	2.10	77
7	1	CL, 1 : 250	24	100	7270	2.22	100
8	1	VL, 1 : 250	24	100	5530	2.07	82
9	1	CL, 1 : 750	24	99	5840	2.55	100
10	1	VL, 1 : 750	24	97	6760	2.14	83

a All reactions were conducted as melts at 130 °C under air.

b 1 mmol of [Cat] corresponds to 1 mmol of 1 or 2, and 2 mmol of 3, (3 contains one zinc centre whilst 1 and 2 contain two cobalt/manganese centres).

c Conversion confirmed by ^1^H NMR spectroscopy.

d GPC analysis in THF at ambient temperature and polystyrene standards were used to calibrate the results (PCL corrected by a factor^27^ of 0.56, PVL corrected by a factor ref. 27 of 0.57).

e Polydispersity index (*M*_w_/*M*_n_) calculated by GPC.

f Determined by weight of resulting polymers.

**Table 2:** ROP of ε-CL or δ-VL initiated by 1–3 under nitrogen^a^

Entry	Catalyst	[Cat] : [monomer]^b^	Time (h)	Conv.^c^ (%)	*M*_n_ d (Da)	*Đ* e	Yield^f^ (%)
1	1	CL, 1 : 500	24	3	—	—	—
2	1	VL, 1 : 500	24	51	7200	1.64	100
3	2	CL, 1 : 500	24	0	—	—	—
4	2	VL, 1 : 500	24	62	3860	1.18	79
5	3	CL, 1 : 500	24	22	2910	1.14	100
6	3	VL, 1 : 500	24	61	9190	1.25	100

a All reactions were as melt, at 130 °C under nitrogen.

b 1 mmol of [Cat] corresponds to 1 mmol 1 or 2, and 2 mmol of 3 (each molecule of 3 contains one zinc centre while each molecule of 1 and 2 contains two cobalt or manganese centres respectively).

c Conversion was confirmed by ^1^H NMR spectroscopy.

d GPC analysis in THF at ambient temperature and polystyrene standards were used to calibrate the results (PCL was corrected by a factor^27^ of 0.56, PVL was corrected by a factor ref. 27 of 0.57).

e Polydispersity index (*M*_w_/*M*_n_) was calculated by GPC.

f Determined by weight of resulting polymers.

In Table S2,† the performance of the systems reported herein is compared against a number of literature catalysts. In terms of conversions, 1–3 perform well *versus* other MOFs though the ZIFs selected seem to be superior. In terms of the molecular weight of the products obtained, the ZIFs highlighted afforded higher molecular weight polymers, whereas the other MOFs selected tended to afford lower molecular polymers. A number of other molecular catalysts are listed in Table S2† which tend to afford lower conversion but higher molecular weights.

### Kinetics studies

**Fig. 7:**
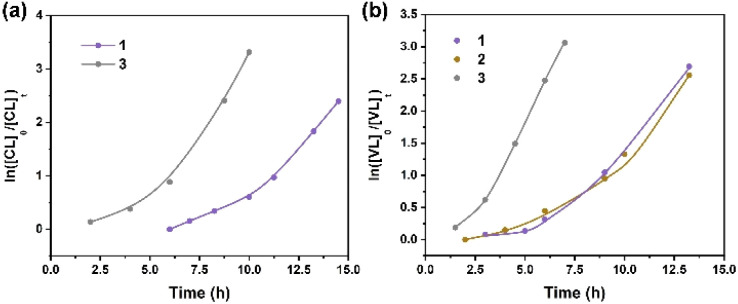
(a) Plot of ln([CL]_0_/[CL]_*t*_) *vs.* time for the polymerization of ε-CL initiated by 1 and 3; (b) plot of ln([VL]_0_/[VL]_*t*_) *vs.* time for the polymerization of δ-VL initiated by 1–3.

**Scheme 2:**
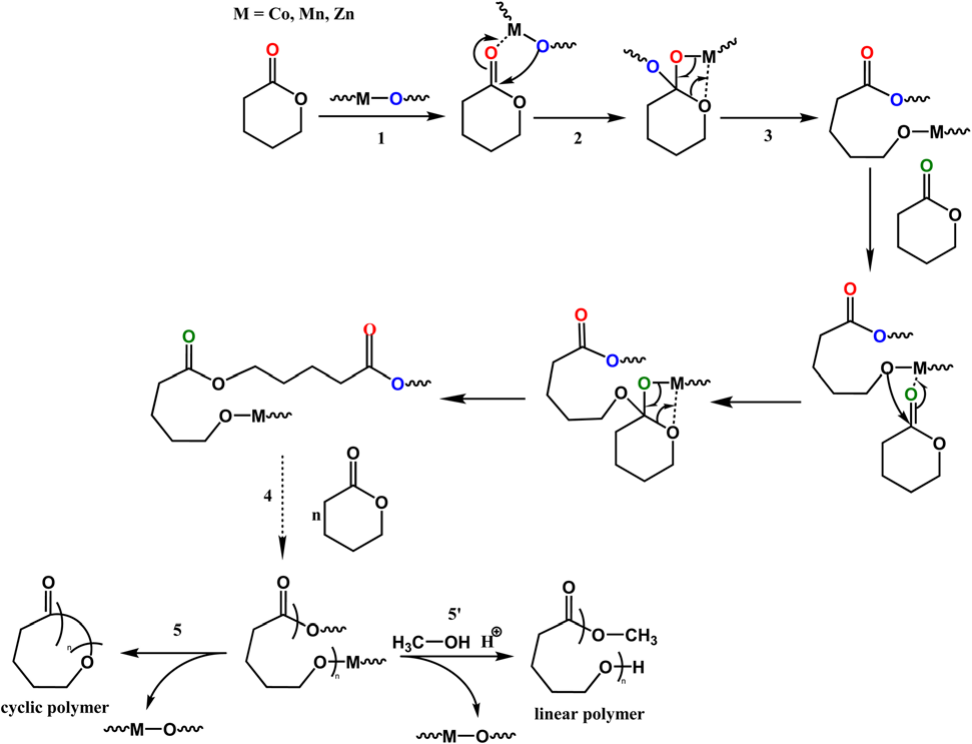
Coordination-insertion mechanism for the ROP of δ-VL initiated by 1–3 and the polymer formation.

Kinetics studies of ε-CL polymerization initiated by 1 and 3 (data for 2 are not shown because of its slow reactivity) are shown in Fig. 7a. Additionally, the plot of ln([VL]_0_/[VL]_*t*_) *vs.* time for the polymerization of δ-VL initiated by 1–3 are presented in Fig. 7b. The observed reactivity trend in both the ε-CL and δ-VL plots is 3 > 1 > 2. Interestingly, an induction period of approximately 3 to 10 h resulted in a curve in the plots. We have previously reported a similar phenomenon, which is attributed to the slow insertion of the CL or VL unit.^28^ The widely accepted mechanism of the ROP of cyclic esters is the “coordination-insertion” mechanism.^29^Scheme 2 gives an example of ROP with δ-VL initiated by 1–3 (this applies to all cyclic esters). There are mainly 5 steps: (1) coordination, whereby the unsaturated metal centre coordinates with the carbonyl oxygen of the monomer; (2) insertion, the oxo ligand of the catalyst inserts into the carbonyl carbon *via* nucleophilic attack; (3) ring opening by the disconnection of the acyl–oxygen bond; (4) chain growth, step 1 to 3 repeated with the addition of monomer; (5) intramolecular transesterification resulting in cyclic polymer and (5′) transesterification by quenching in acidic methanol resulting in a linear polymer. As a type of coordination compound, MOFs also follow this mechanism. However, steric effects cannot be ignored in these MOFs with their small interior cavities, as has been reported in previous literature.^30^ Based on observations from TGA tests (Fig. S26†), only solvent of crystallization is lost for 1–3 prior to reaching the temperature employed for the ROP. Only at temperatures in excess of 200 °C is significant decomposition observed.

### Recyclability studies

**Fig. 8:**
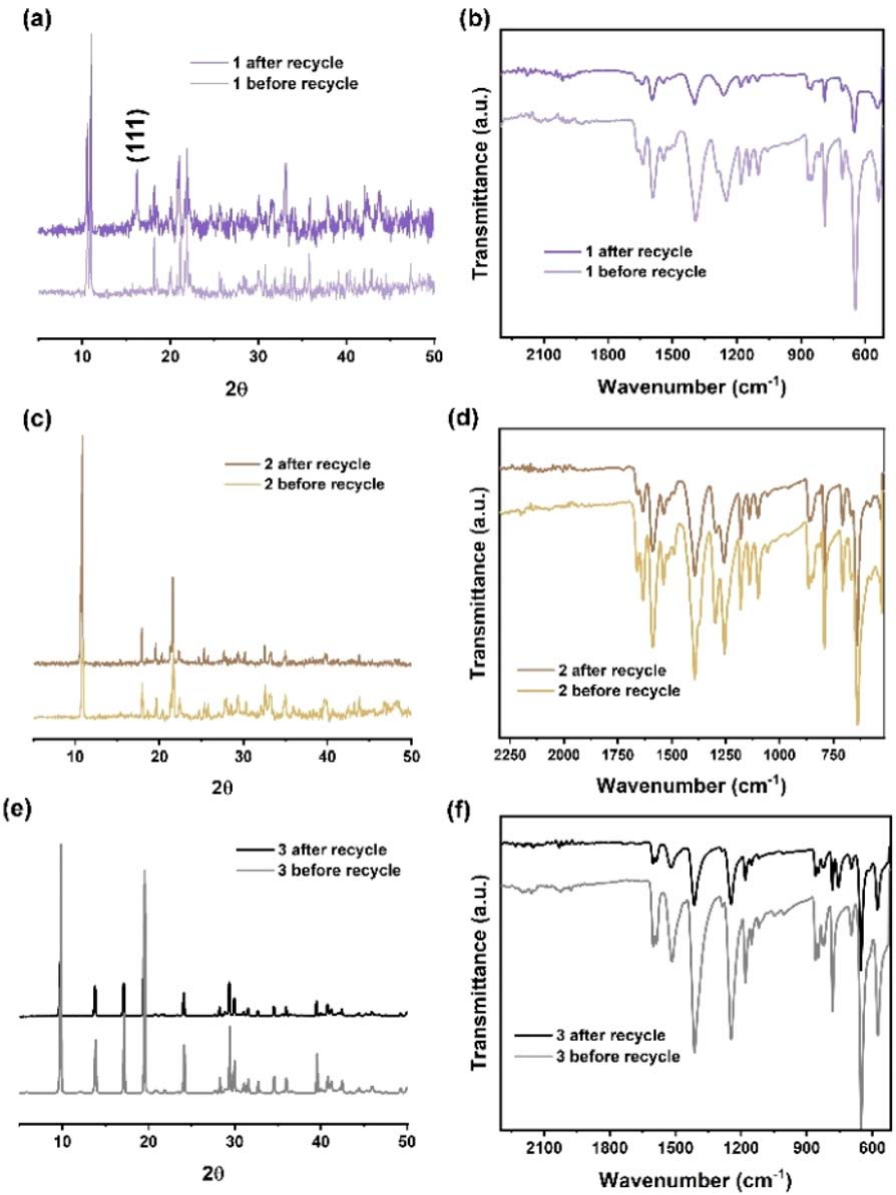
(a) X-ray powder diffraction patterns; (b) infrared spectra of 1, before and after recycling; (c) X-ray powder diffraction patterns; (d) infrared spectra of 2, before and after recycling; (e) X-ray powder diffraction patterns; (f) infrared spectra of 3, before and after recycling.

To investigate the stability of the catalysts during the ROP reaction, recyclability studies were performed. 1 was collected and separated from the resulting PCL after reaction (conducted at 130 °C for 24 h in the melt state under air). The PXRD pattern (Fig. 8a) of 1 before and after recycling indicates that a small portion of 1 was oxidized during the ROP reaction. This oxidation is confirmed by the presence of peaks corresponding to the (111) crystal plane of Co_3_O_4_ (ref. 31) in the PXRD pattern of 1 after recycling. The IR spectra of 1 before and after recycling (Fig. 8b) are consistent, suggesting that the ligand set remains stable during the ROP reaction. In addition, the PXRD patterns and IR spectra of 2 and 3 before and after recycling indicate that the manganese and zinc MOFs retain their structures following the ROP reaction.

## Conclusions

Use of *p*-hydroxybenzoic acid in solvothermal syntheses involving either M(OAc)_2_·4H_2_O (M = Co or Mn) and DMF, results in the 3D MOFs {[Co(O_2_CC_6_H_4_O)(DMF)]_2_}_*n*_ (1) and {[Mn(O_2_CC_6_H_4_O)(DMF)]_2_}_*n*_ (2) being isolated in good yields (80 and 62%, respectively). Both 1 and 2 have been structurally characterized. ROP studies involving 1 and 2, together with the known zinc MOF Zn(O_2_CC_6_H_4_O) (3), on ε-CL and δ-VL indicated the formation of mostly cyclic polymers, and the catalytic rate 3 > 1 > 2. Recyclability tests demonstrated that 1–3 exhibit stability with only partial oxidation of 1 observed after repeated ROP cycles.

## Data availability

The crystallographic data herein has been deposited in the Cambridge Structural Database, see numbers 2375107 and 2375108.

## Conflicts of interest

There are no conflicts to declare.

## Supplementary Material

RA-014-D4RA06361A-s001

RA-014-D4RA06361A-s002
