# Supplementary material for: Metal–organic frameworks of p-hydroxybenzoic acid: synthesis, structure and ring opening polymerization capability
Source: RSC Adv. 2024 Nov 27;14(51):37895–901. doi: 10.1039/d4ra06361a (PMC11600306; doi:10.1039/d4ra06361a)
Supplement: RA-014-D4RA06361A-s001 [file RA-014-D4RA06361A-s001.pdf]

Supplementary Information (SI) for RSC Advances.
This journal is © The Royal Society of Chemistry 2024

Metal-organic frameworks of *p*-hydroxybenzoic acid: synthesis, stucture and ring opening polymerization capabity.

Yi Gong,[a] Simon Sharp,[b] Mark R.J. Elsegood[b] and Carl Redshaw *[a]

[a] *Chemistry, School of Natural Sciences, University of Hull, Hull, HU6 7RX, U.K. E-mail: C.Redshaw@hull.ac.uk*
[b] *Chemistry Department, Loughborough University, Loughborough, LE11 3TU, U.K. E-mail: xxxx@aaa.bbb.ccc*

Table of contents

Table S1. Crystal data and structure refinement for **1** and **2**.
Figure S1. X-ray powder diffraction pattern of **3** and its simulation.
Figure S2. Infrared spectra of **1** – **3**.
Figure S3. Alternative view of the 'building block' in **1**.
Figure S4. Side view of channel in **1** showing how the aromatic groups block access through sides of the channel (*c* axis).
Figure S5. Alternative view of the 'building block' of **2**.
Figure S6. Side view of channels in **2**.
Single crystal X-ray diffraction experimental.
Figure S7. ^{1}H NMR spectrum of PCL in $CDCl_3$ at 298 K (Table 1, entry 1).
Figure S8. ^{1}H NMR spectrum of PVL in $CDCl_3$ at 298 K (Table 1, entry 2).
Figure S9. ^{1}H NMR spectrum of PCL in $CDCl_3$ at 298 K (Table 1, entry 3).
Figure S10. ^{1}H NMR spectrum of PVL in $CDCl_3$ at 298 K (Table 1, entry 4).
Figure S11. ^{1}H NMR spectrum of PCL in $CDCl_3$ at 298 K (Table 1, entry 5).
Figure S12. ^{1}H NMR spectrum of PVL in $CDCl_3$ at 298 K (Table 1, entry 6).
Figure S13. MALDI-TOF mass spectrum of PCL (Table 1, entry 1).
Figure S14. MALDI-TOF mass spectrum of PCL (Table 1, entry 3).
Figure S15. MALDI-TOF mass spectrum of PVL (Table 1, entry 4).
Figure S16. MALDI-TOF mass spectrum of PVL (Table 1, entry 6).
Figure S17. ^{1}H NMR spectrum of PCL in $CDCl_3$ at 298 K (Table 2, entry 5).
Figure S18. ^{1}H NMR spectrum of PVL in $CDCl_3$ at 298 K (Table 2, entry 2).
Figure S19. ^{1}H NMR spectrum of PVL in $CDCl_3$ at 298 K (Table 2, entry 4).
Figure S20. ^{1}H NMR spectrum of PVL in $CDCl_3$ at 298 K (Table 2, entry 6).
Figure S21. MALDI-TOF mass spectrum of PVL (Table 2, entry 2).
Figure S22. MALDI-TOF mass spectrum of PVL (Table 2, entry 4).
Figure S23. MALDI-TOF mass spectrum of PVL (Table 2, entry 6).
Figure S24. GPC trace of (a) PCL from table 1, entry 1, 3, 5 and (b) PVL from table 1, entry 2, 4, 6.
Figure S25. GPC trace of PVL from table 2, entry 2, 4, 6.
Figure S26. Thermogravimetric pattern of **1** and **2**.
Table S2. Comparison with other complexes.
References

Table S1. Crystal data and structure refinement for **1** and **2**.

	**1**	**2**
Chemical formula	$C_{20}H_{22}Co_2N_2O_8$	$C_{20}H_{22}Mn_2N_2O_8$
Formula weight	536.25	528.27
Temperature	120(2) K	100(2) K
Radiation, wavelength	Mo*K*α, 0.71073 Å	Cu*K*α, 1.54178 Å
Crystal system, space group	orthorhombic, *Pbca*	orthorhombic, *Pbca*
Unit cell parameters	a = 16.7720(4) Å; α = 90°<br>b = 12.6914(2) Å; β = 90°<br>c = 18.7308(4) Å; γ = 90°	a = 16.6962(5) Å; α = 90°<br>b = 13.0537(4) Å; β = 90°<br>c = 19.1273(9) Å; γ = 90°
Cell volume	3987.04(14) $Å^3$	4168.7(3) $Å^3$
Z	8	8
Calculated density	1.787 g/cm^3	1.683 g/cm^3
Absorption coefficient μ	1.72 mm^{-1}	10.32 mm^{-1}
F(000)	2192	2160
Crystal colour and size	intense blue, 0.14 × 0.08 × 0.04 mm^3	colourless plate, 0.05 × 0.03 × 0.02 mm^3
Reflections for cell refinement	34638	42613
θ range for data collection	2.9 to 27.5°	4.6 to 73.7°
Index ranges	$h = -21 \rightarrow 21$, $k = -15 \rightarrow 16$, $l = -24 \rightarrow 24$	$h = -20 \rightarrow 20$, $k = -15 \rightarrow 14$, $l = -23 \rightarrow 23$
Completeness to $\theta = 27.5°$	99.8 %	99.9 %
Reflections collected	32878	42613
Independent reflections	4572 (R_{int} = 0.057)	4064 (R_{int} = 0.079)
Reflections with F^2>2σ	3918	3129
Absorption correction	semi-empirical from equivalents	analytical
Min. and max. transmission	0.795 and 0.935	0.845 and 0.902
Refinement method	Full-matrix least-squares on F^2	Full-matrix least-squares on F^2
Weighting parameters a, b	0.0592, 39.6136	0.1035, 9.7771
Data / restraints / parameters	4572 / 369 / 348	4064 / 578 / 402
*R*1 [F^2>2σ]	0.071,	0.058
*wR*2 (all data)	0.179	0.172
Goodness-of-fit on F^2	1.10	1.04
Largest diff. peak and hole	1.71 and –1.25 e $Å^{-3}$	1.12 and –0.71 e $Å^{-3}$

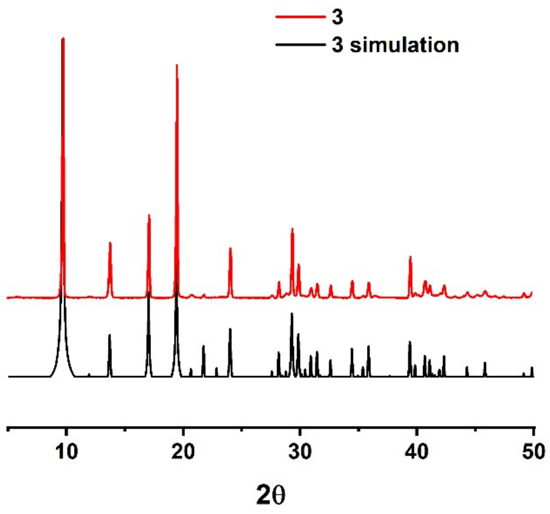

Figure S1. X-ray powder diffraction pattern of **3** and its simulation.

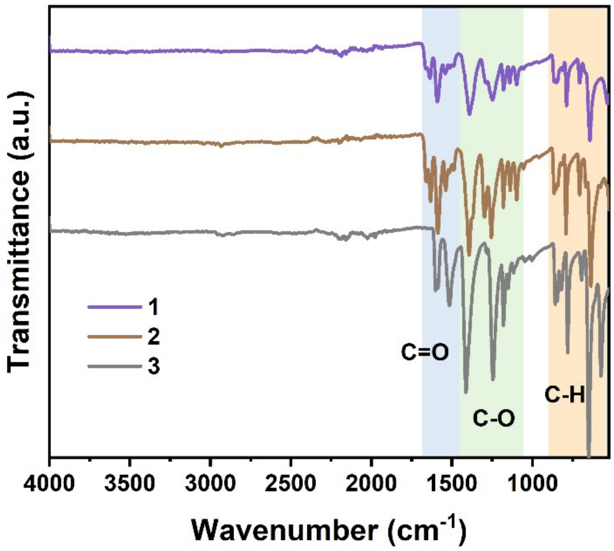

Figure S2. Infrared spectra of **1** – **3**.

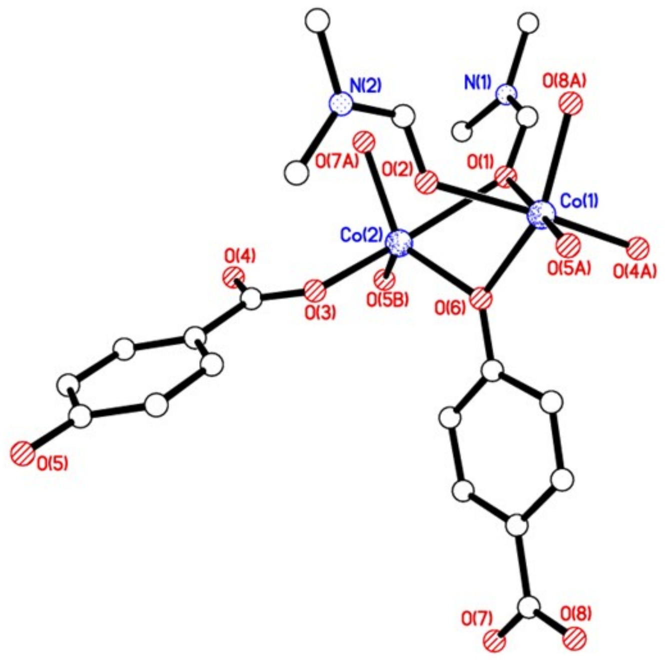

Figure S3. Alternative view of the ‘building block’ in **1**.

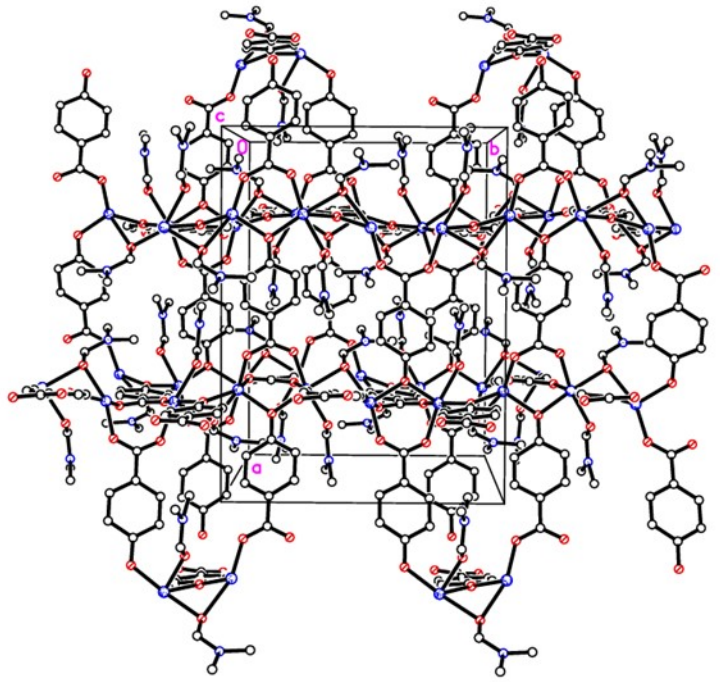

Figure S4. Side view of channel in **1** showing how the aromatic groups block access through sides of the channel (*c* axis).

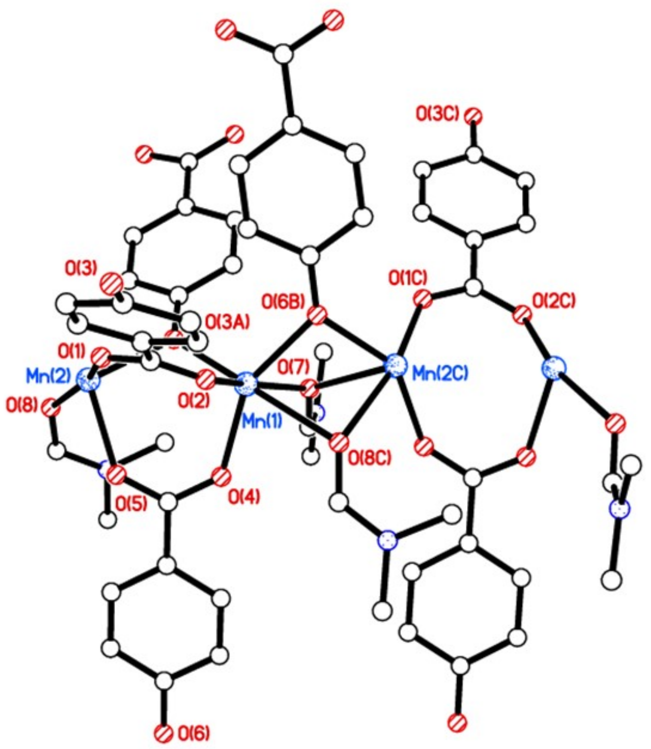

Figure S5. Alternative view of the ‘building block’ of **2**.

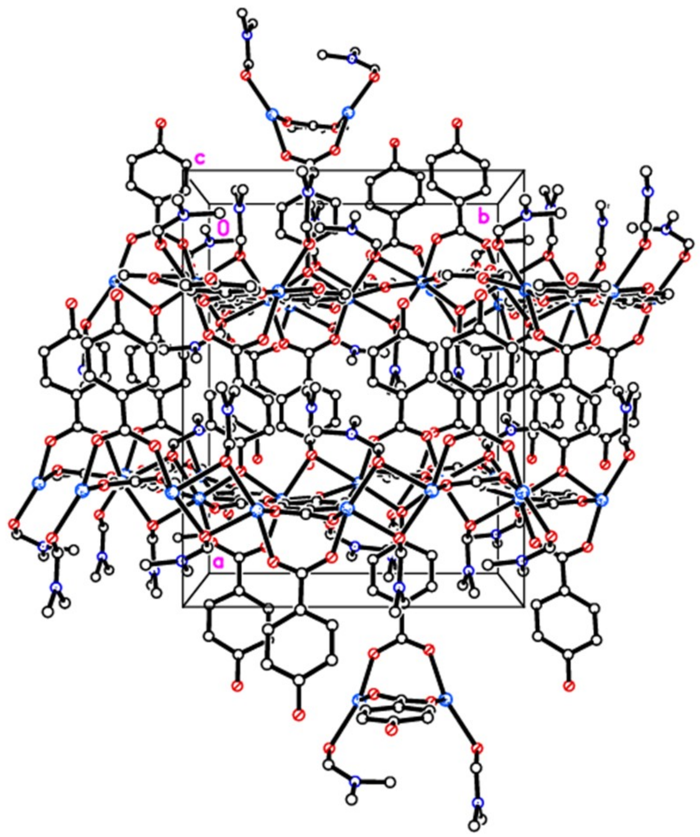

Figure S6. Side view of filled channels in **2**.

Single Crystal X-ray diffraction experimental.

Diffraction data were collected on a Bruker-Nonius APEX II CCD diffractometer [1,2] for **1** and a Rigaku 007HF diffractometer equipped with a HyPix Arc-100 detector for **2**. The data were corrected for absorption and Lp effects. Full details are given in Table S1 above. The two structures are close to isomorphic with the Mn structure having the slightly larger unit cell volume. In both cases one acid molecule, containing atom O(4), was modelled as two-fold disordered with major occupancy 73.6(16) for **1**, where atoms C(12) > C(14) & O(6) were common to both components, and 95.2(2)% for **2**, where the whole ligand and the two metal ions were modelled as disordered. The structures were solved by direct methods [4] for **1** and via a charge flipping algorithm for **2** [5] and refined on F^2 values [6].

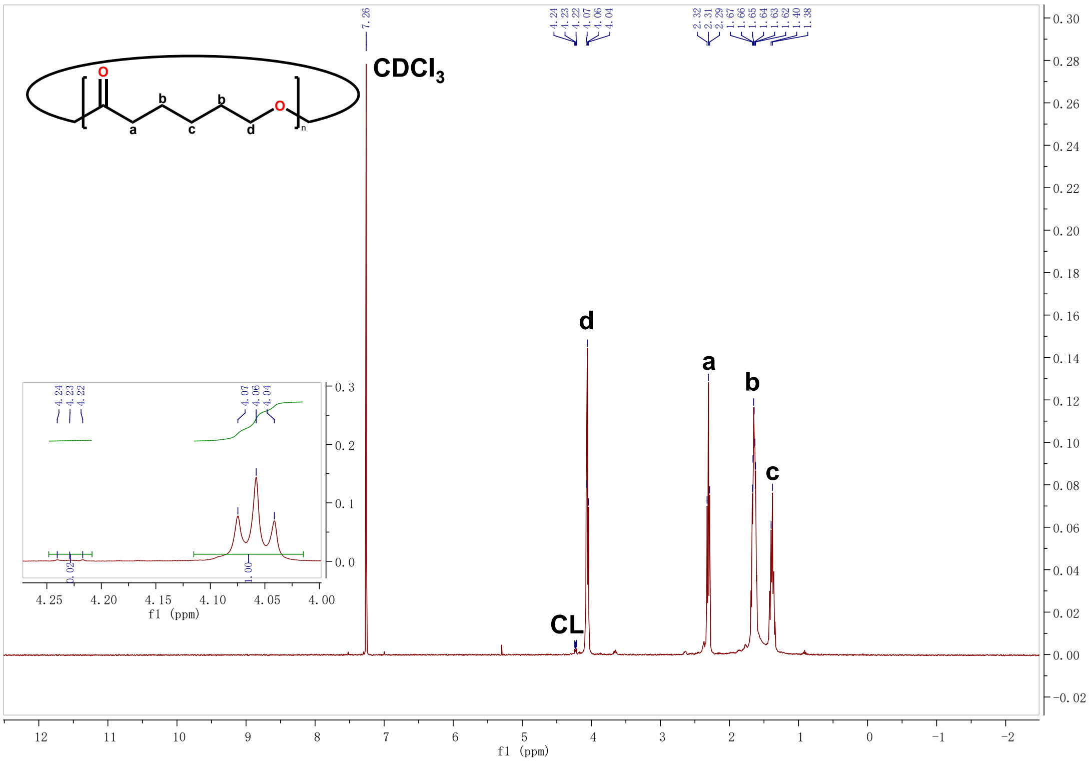

Figure S7. ^{1}H NMR spectrum of PCL in $CDCl_3$ at 298 K (Table 1, entry 1).

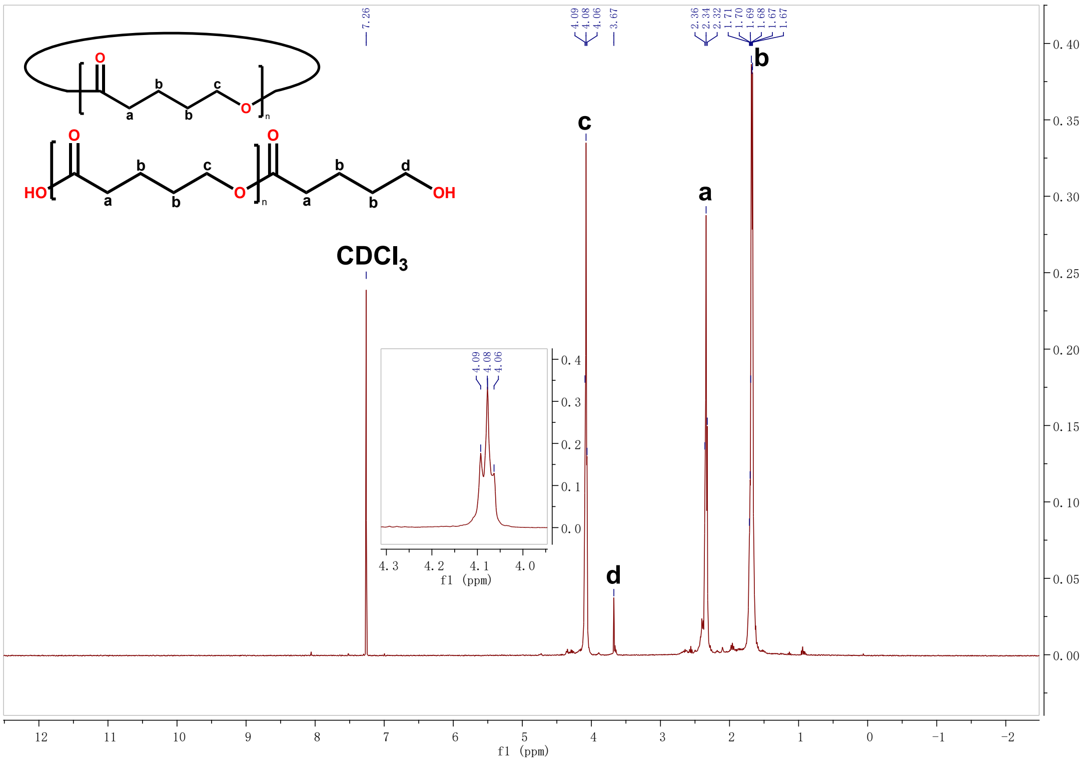

Figure S8. ^{1}H NMR spectrum of PVL in $CDCl_3$ at 298 K (Table 1, entry 2).

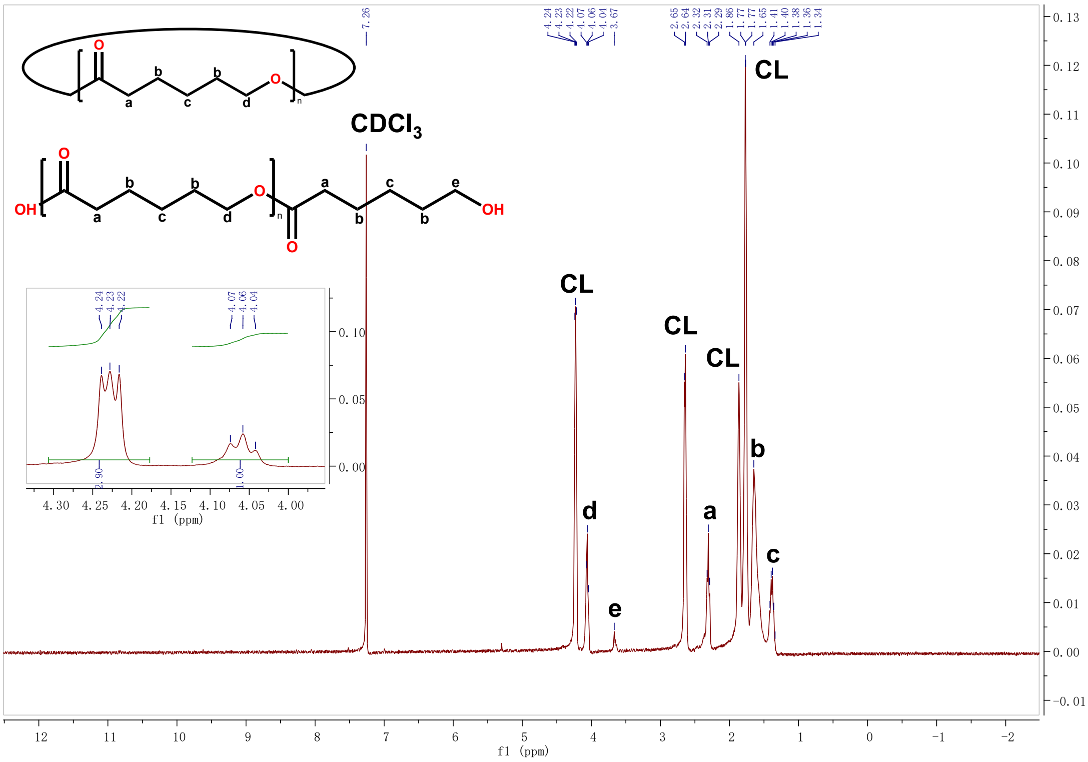

Figure S9. ^1H NMR spectrum of PCL in $CDCl_3$ at 298 K (Table 1, entry 3).

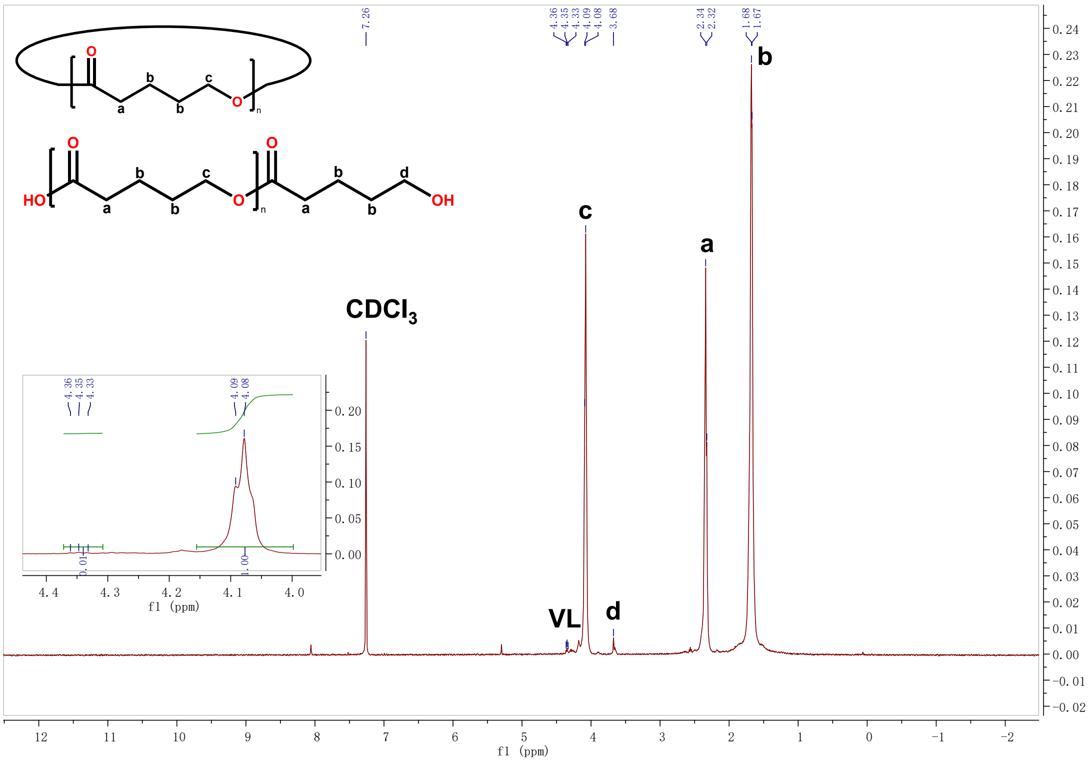

Figure S10. ^1H NMR spectrum of PVL in $CDCl_3$ at 298 K (Table 1, entry 4).

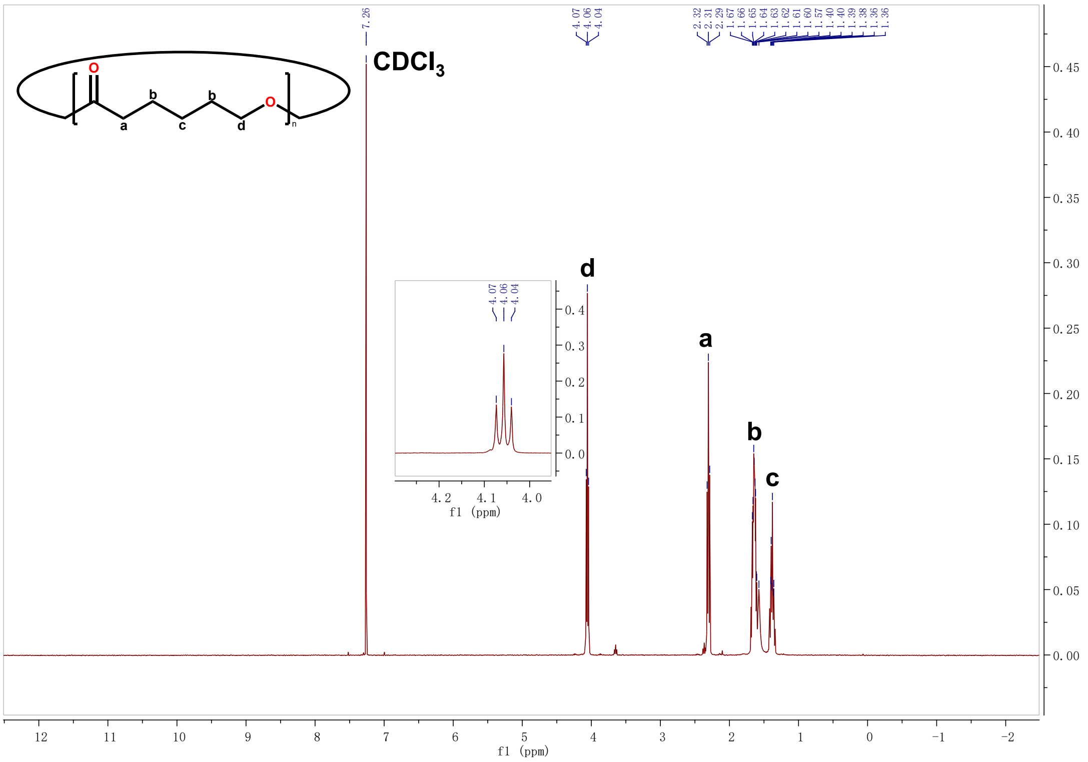

Figure S11. ^{1}H NMR spectrum of PCL in $CDCl_3$ at 298 K (Table 1, entry 5).

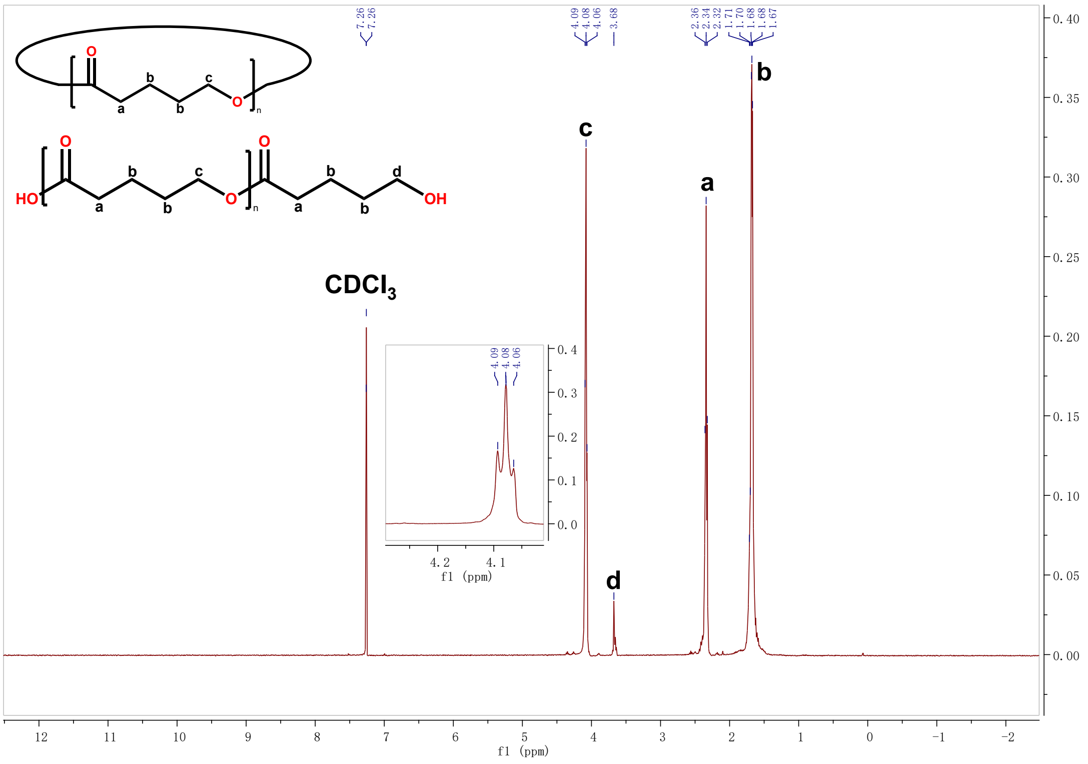

Figure S12. ^{1}H NMR spectrum of PVL in $CDCl_3$ at 298 K (Table 1, entry 6).

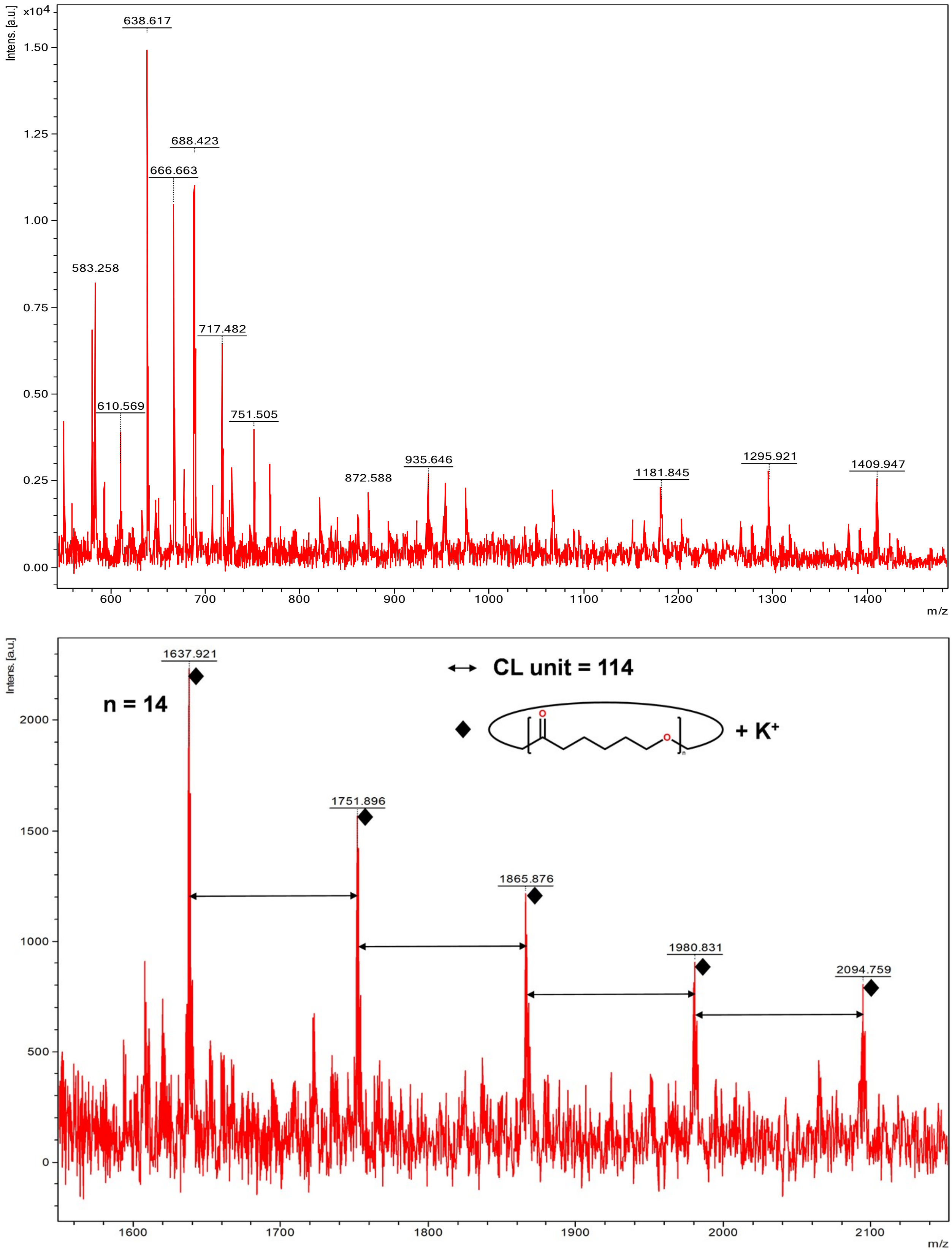

Figure S13. MALDI-TOF mass spectrum of PCL (Table 1, entry 1).

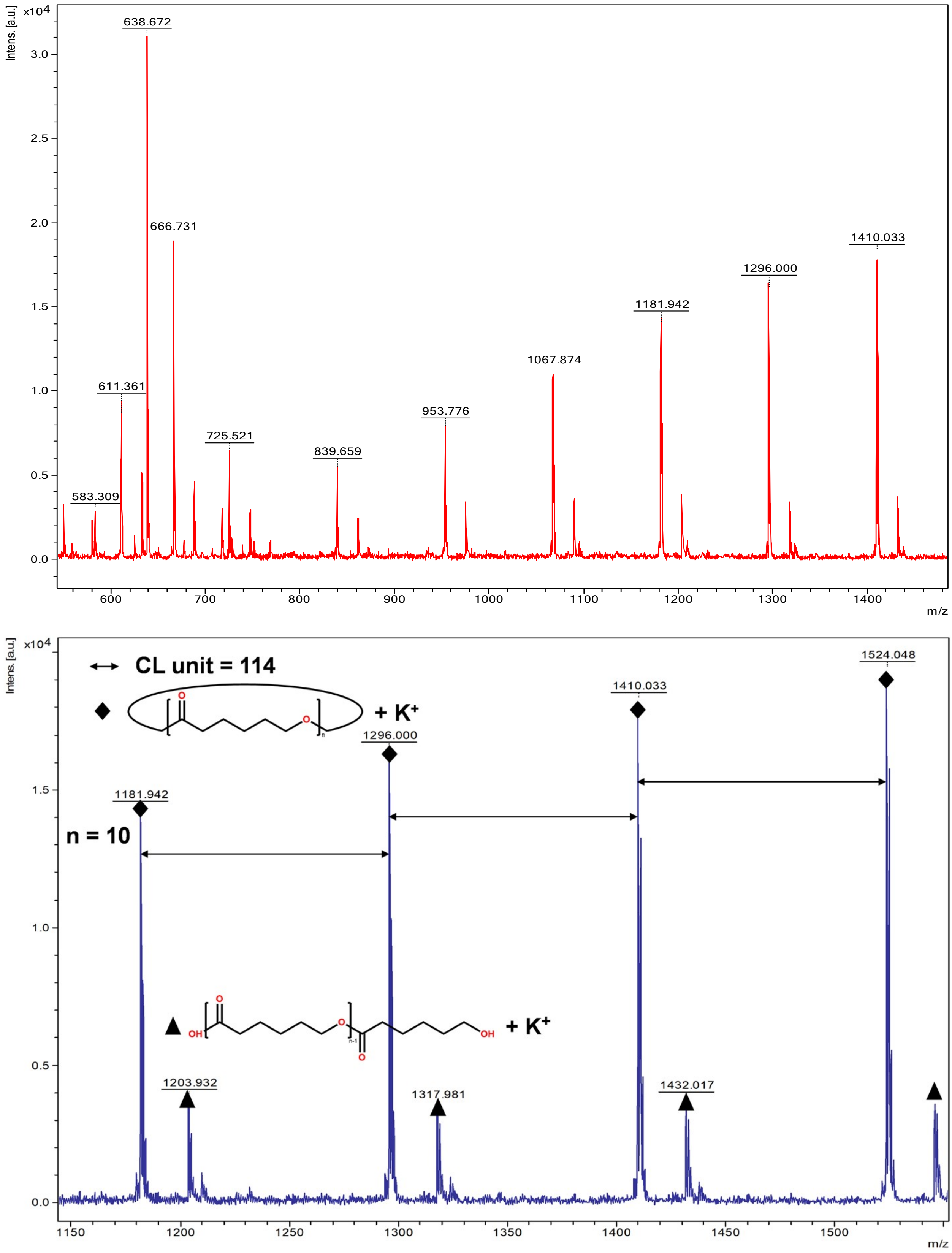

Figure S14. MALDI-TOF mass spectrum of PCL (Table 1, entry 3).

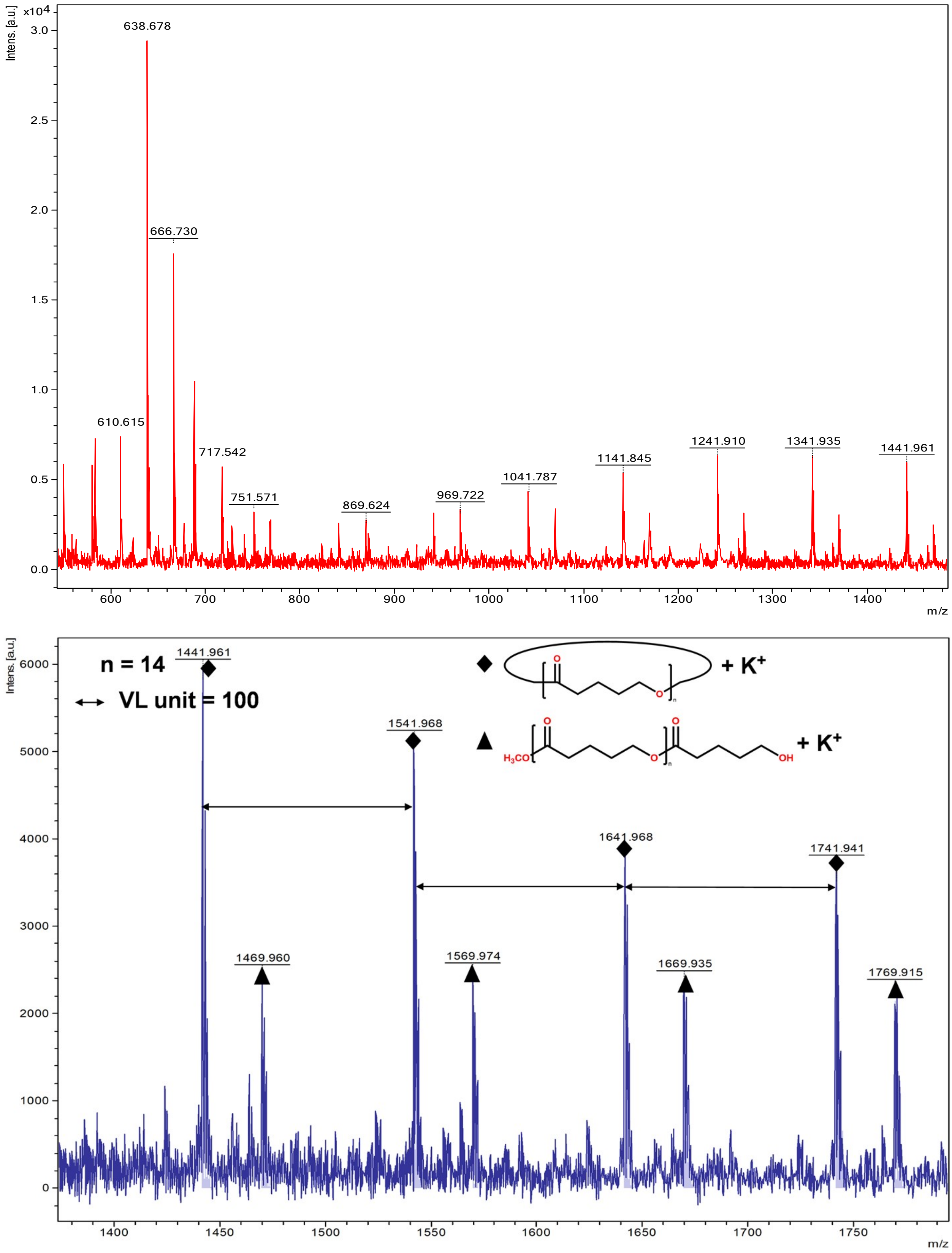

Figure S15. MALDI-TOF mass spectrum of PVL (Table 1, entry 4).

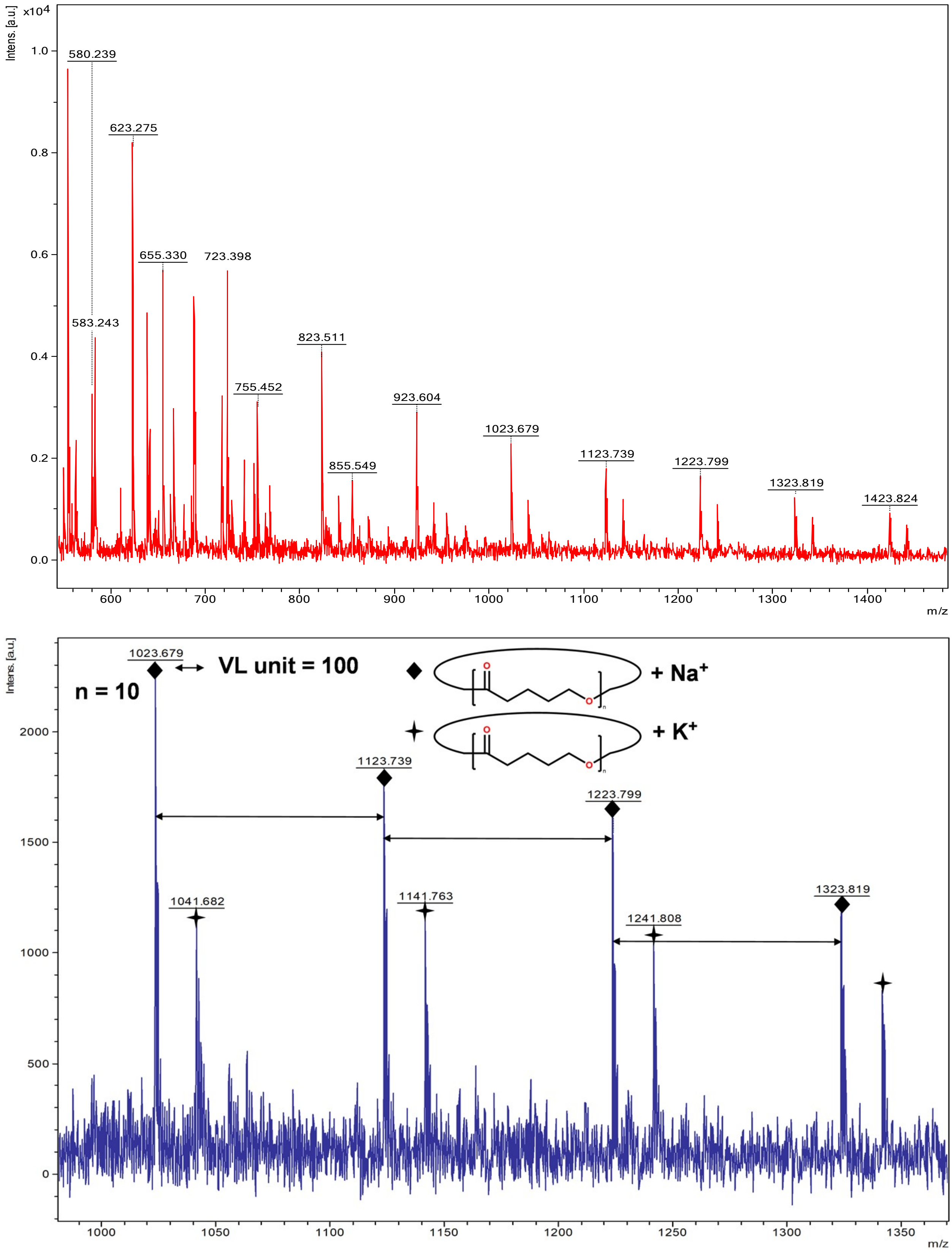

Figure S16. MALDI-TOF mass spectrum of PVL (Table 1, entry 6).

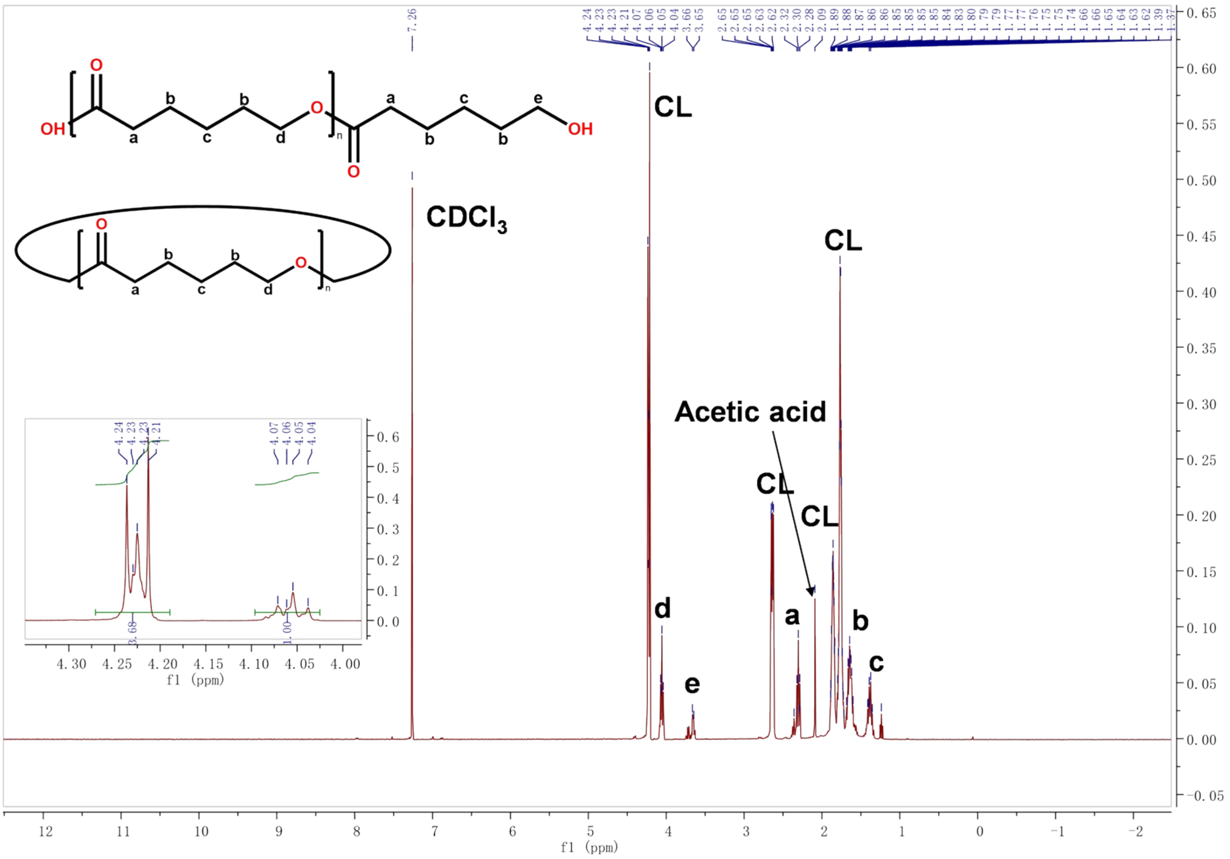

Figure S17. ^{1}H NMR spectrum of PCL in $CDCl_3$ at 298 K (Table 2, entry 5).

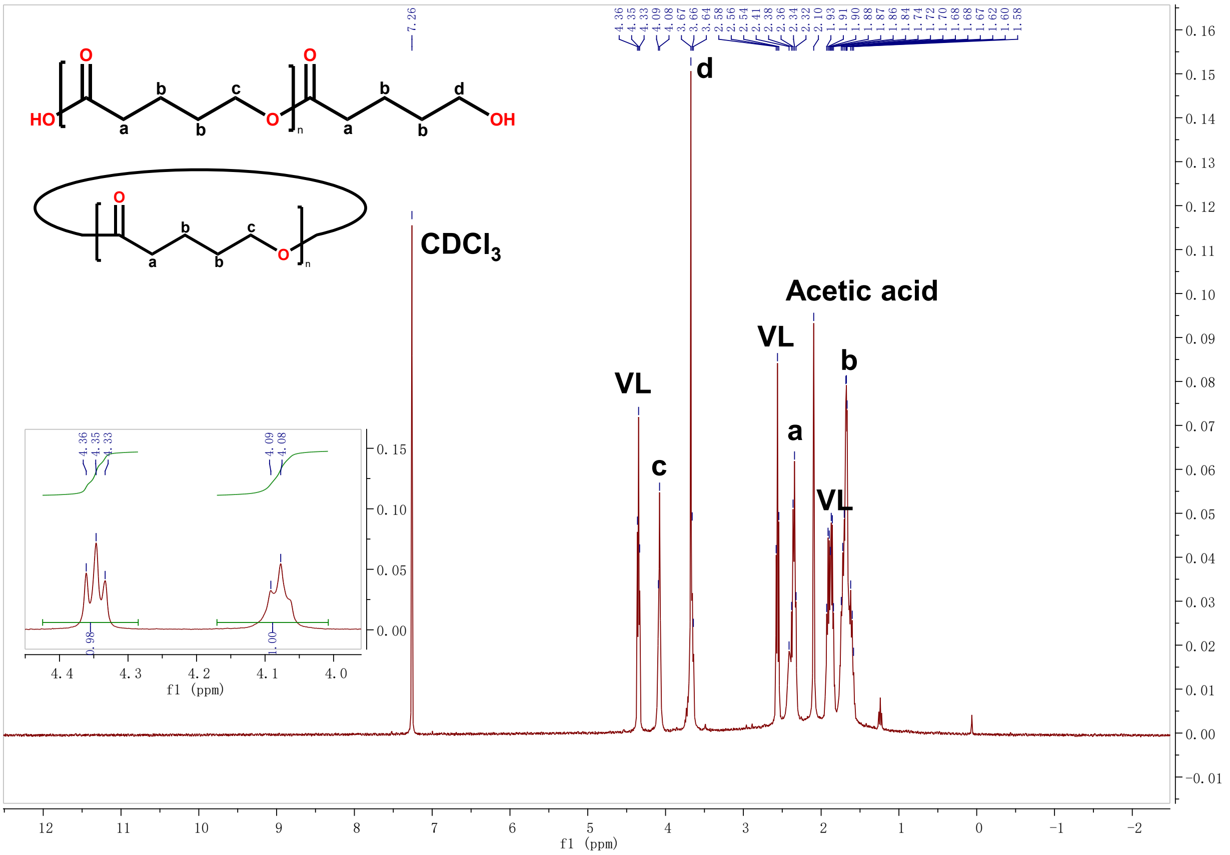

Figure S18. ^{1}H NMR spectrum of PVL in $CDCl_3$ at 298 K (Table 2, entry 2).

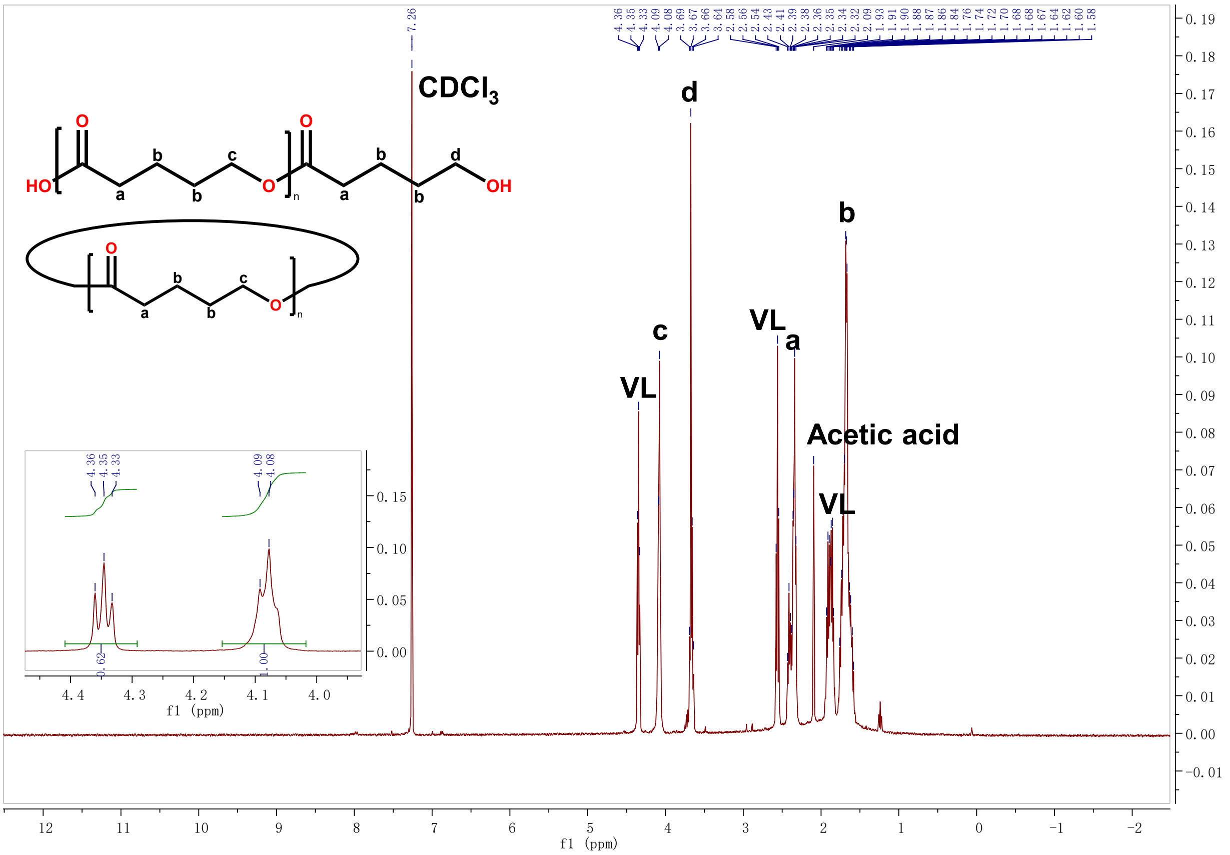

Figure S19. ^{1}H NMR spectrum of PVL in $CDCl_3$ at 298 K (Table 2, entry 4).

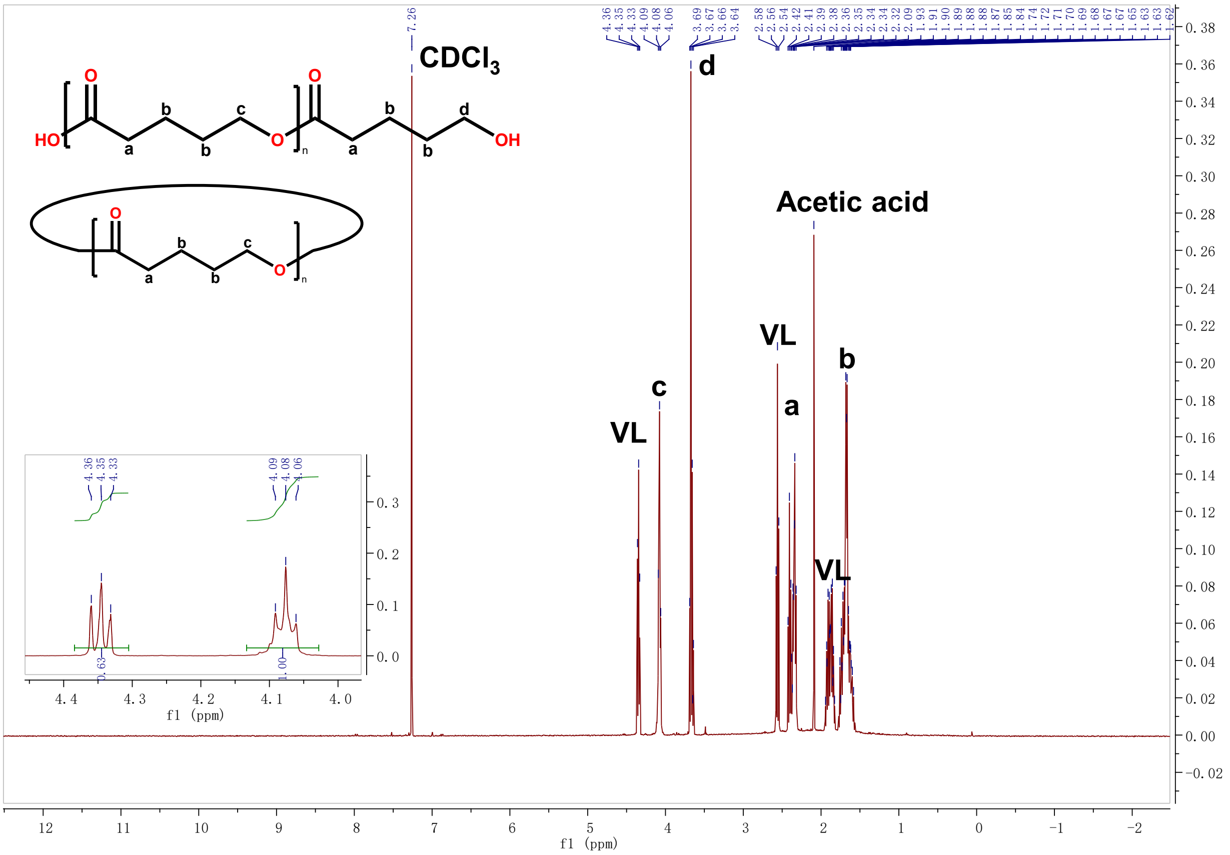

Figure S20. ^{1}H NMR spectrum of PVL in $CDCl_3$ at 298 K (Table 2, entry 6).

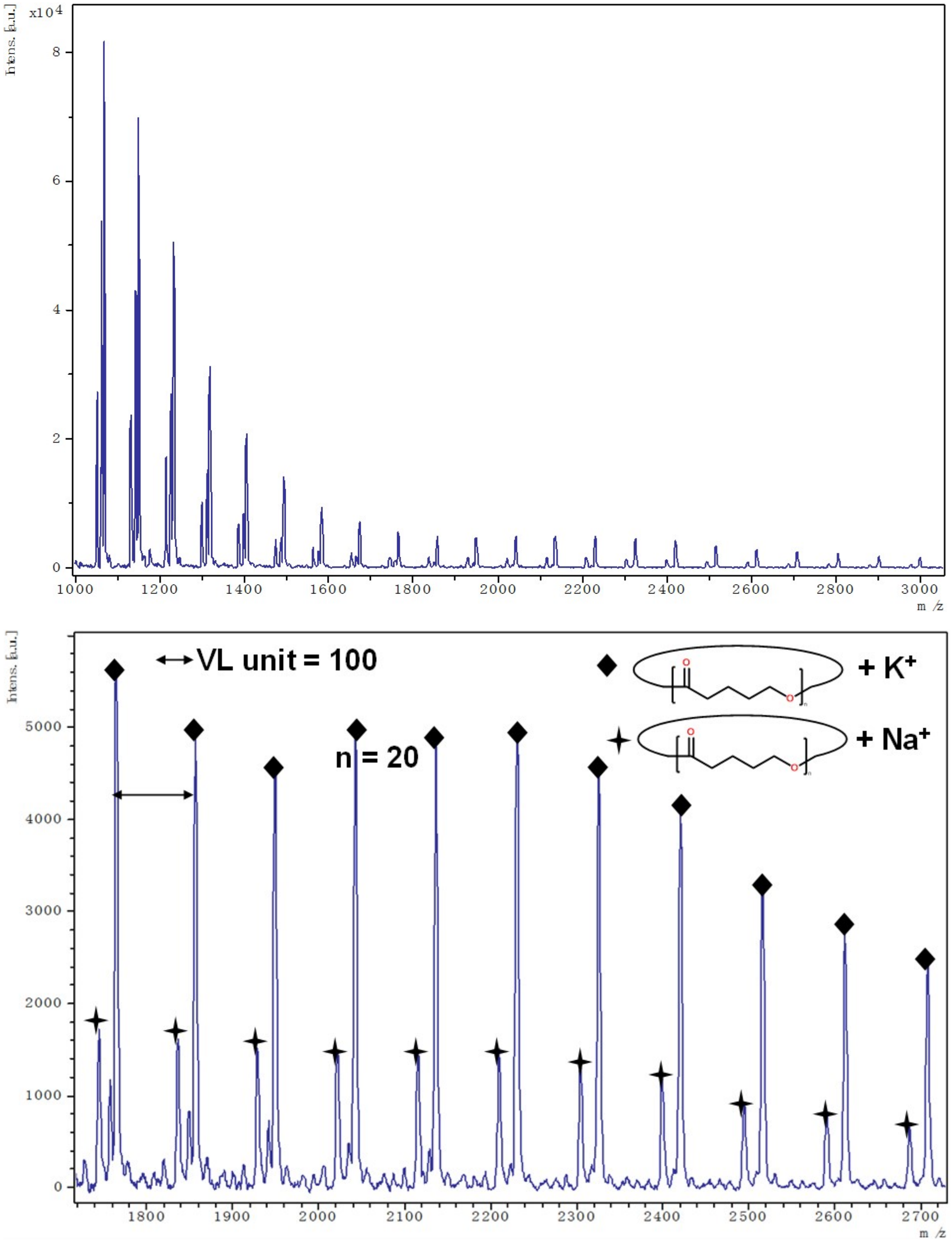

Figure S21. MALDI-TOF mass spectrum of PVL (Table 2, entry 2). Two types of peaks were observed. The peak at *m/z* 2042.888 represents cyclic PVL with K^+ (*m/z*: 100.1 x n + 39.1, n = 20), the peak at *m/z* 2022.125 represents cyclic PVL with Na^+ (*m/z*: 100.1 x n + 23.0, n = 20).

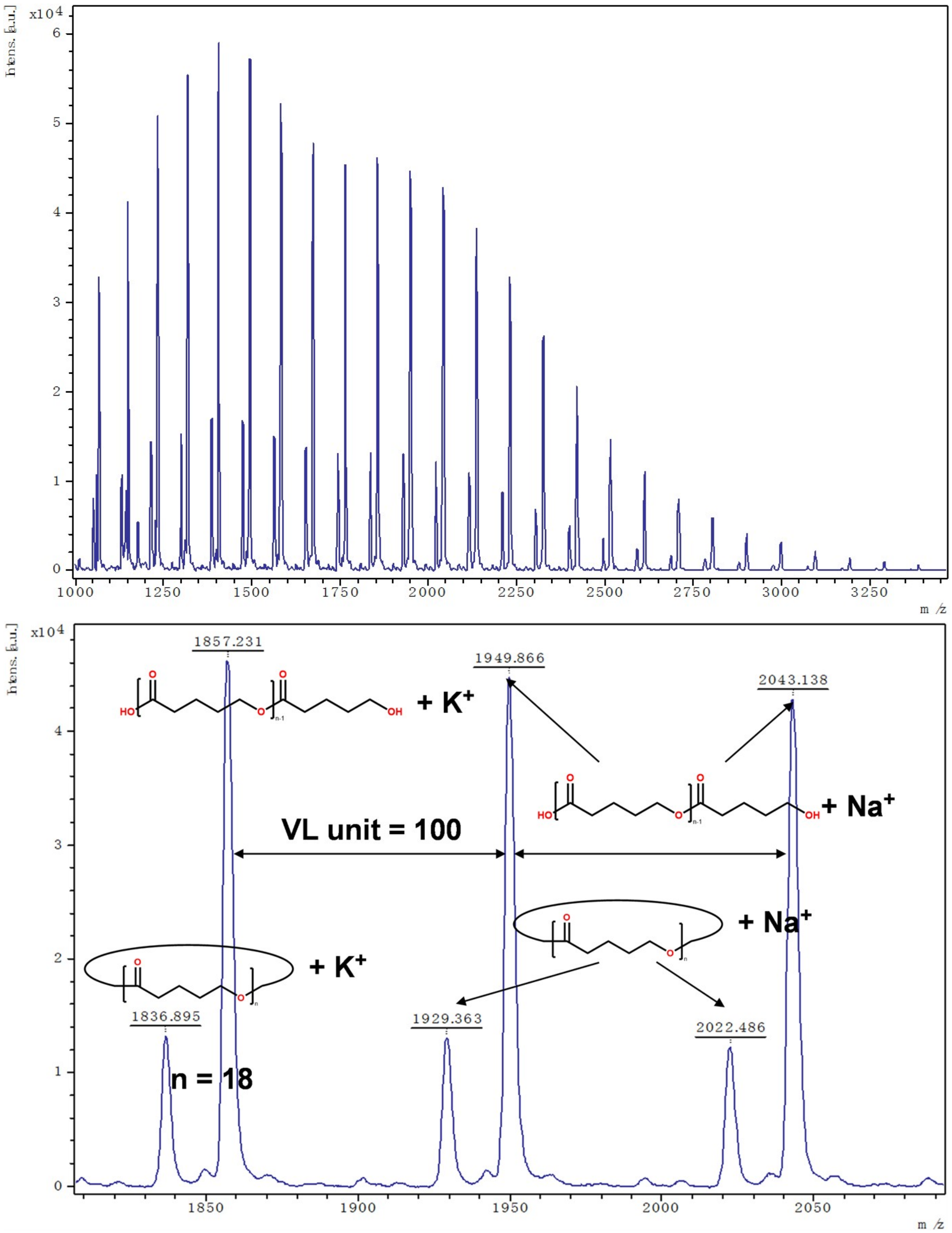

Figure S22. MALDI-TOF mass spectrum of PVL (Table 2, entry 4). The peak at *m/z* 1836.895 represents cyclic PVL with K^+ (*m/z*: 100.1 x n + 39.1, n = 18), the peak at *m/z* 1857.231 represents linear PVL HO(VL)$_n$H with K^+ (*m/z*: 100.1 x n + 18.0 + 39.1, n = 18), the peak at *m/z* 1929.363 represents cyclic PVL with Na^+ (*m/z*: 100.1 x n + 23.0, n = 18), the peak at *m/z* 2043.3214 represents linear PVL HO(VL)$_n$H with Na^+ (*m/z*: 100.1 x n + 23.0, n = 20).

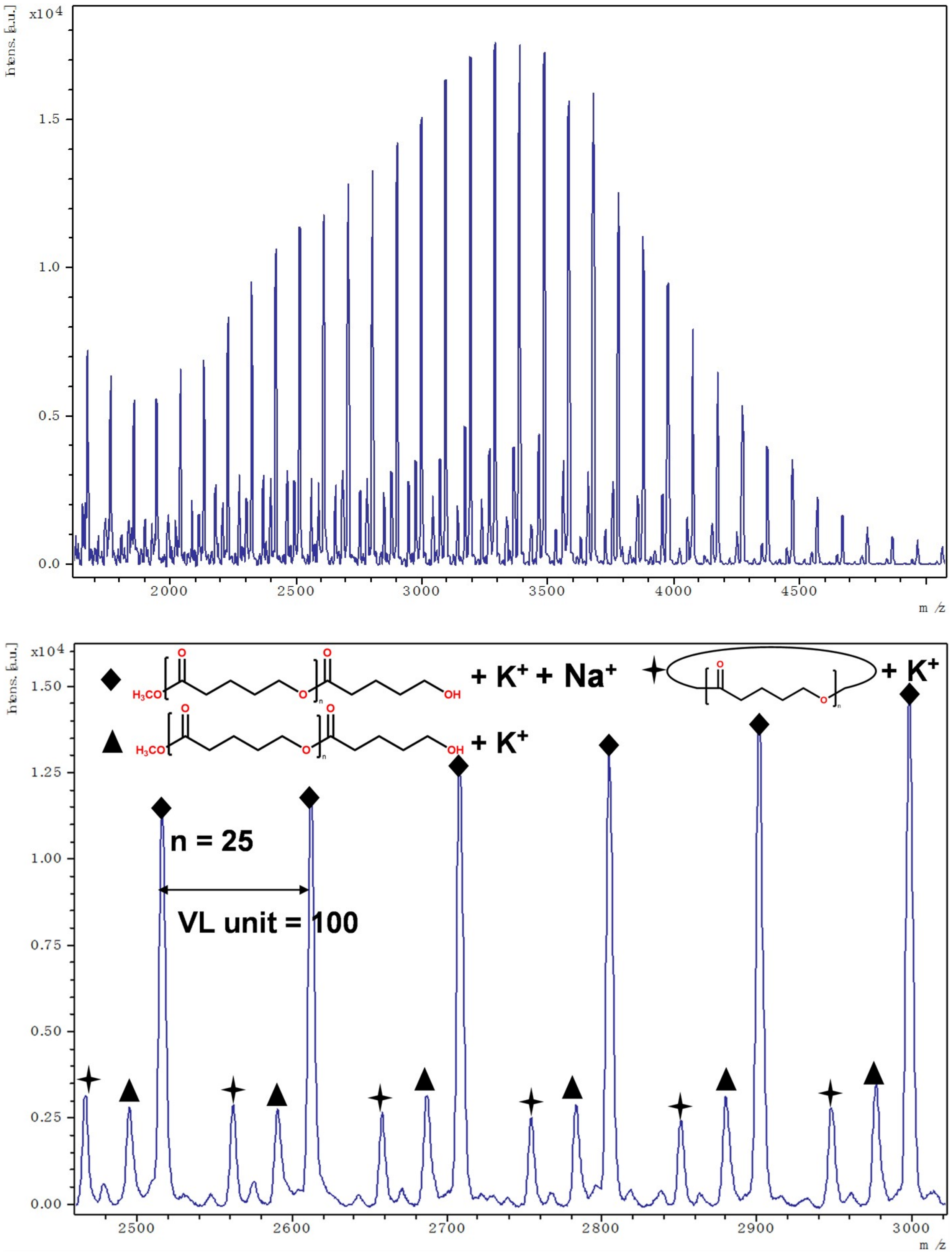

Figure S23. MALDI-TOF mass spectrum of PVL (Table 2, entry 6). Three types of peaks were observed. The peak at *m/z* 3074.6148 represents linear PVL $H_3CO(VL)_nH$ with K^+ (*m/z*: 100.1 x n + 32.0 + 39.1, n = 30), the peak at *m/z* 2999.008 represents linear PVL $H_3CO(VL)_nH$ with K^+ and Na^+ (*m/z*: 100.1 x n + 32.0 + 39.1+ 23.0, n = 29), the peak at *m/z* 3142.373 represents cyclic PVL with K^+ (*m/z*: 100.1 x n + 39.1, n = 31).

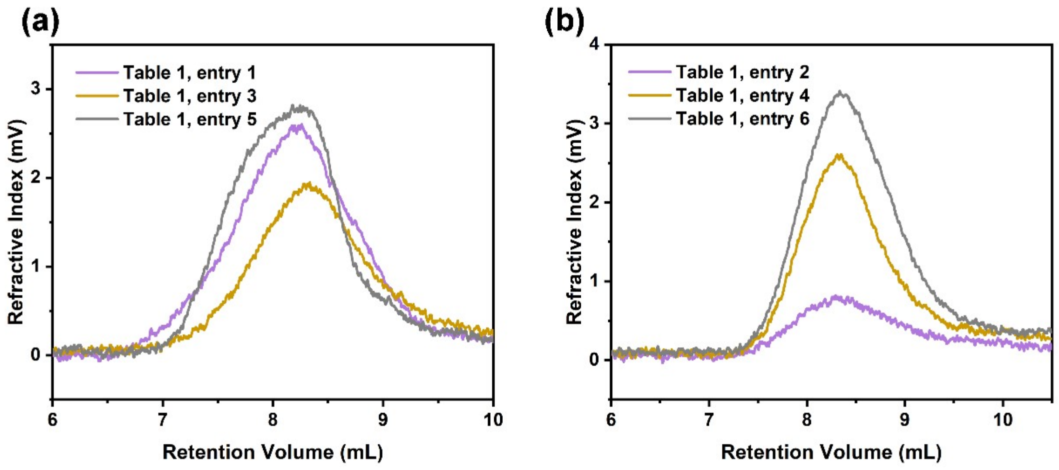

Figure S24. GPC trace of (a) PCL from table 1, entry 1, 3, 5 and (b) PVL from table 1, entry 2, 4, 6.

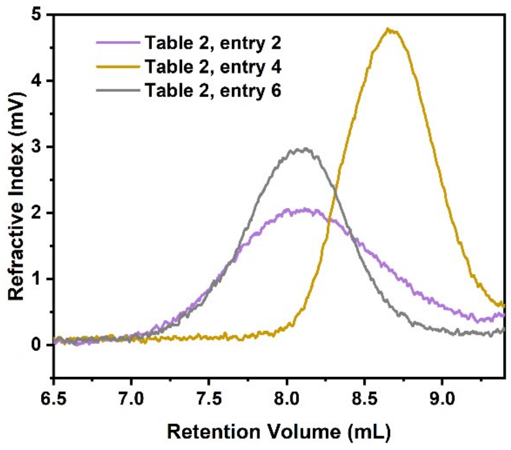

Figure S25. GPC trace of PVL from table 2, entry 2, 4, 6.

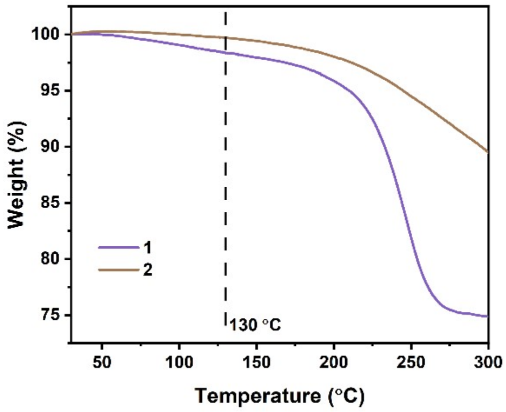

Figure S26. Thermogravimetric pattern of **1** and **2** conducted by PerkinElmer Thermogravimetric Analyzer TGA 4000 (for **3** see the literature[7]).

Table S2. Comparison with other complexes.

Catalyst	Reaction conditions	Monomer and ratio[a]	Conv. (%)	M_n (Da)	*Đ*
**1** (This paper)	130 °C, 24 h, inert atmosphere	[CL]:[Cat] = 500:1	-	-	-
CZU-5[8]	150 °C, 24 h, inert atmosphere	[CL]:[Cat] = 1000:1	56	12800	1.25
**1** (This paper)	130 °C, 24 h, in air	[CL]:[Cat] = 500:1	98	11790	3.04
MOF-74-Co[b]	130 °C, 24 h, in air	[CL]:[Cat] = 500:1	-	-	-
**1** (This paper)	130 °C, 24 h, inert atmosphere	[VL]:[Cat] = 500:1	51	7200	1.64
ZIF-67[7]	140 °C, 4 h, inert atmosphere	[VL]:[Cat] = 20:1	69	17330	1.34
**1** (This paper)	130 °C, 24 h, in air	[VL]:[Cat] = 500:1	100	11440	3.28
MOF-74-Co[b]	130 °C, 24 h, in air	[VL]:[Cat] = 500:1	96	6510	1.41
**2** (This paper)	130 °C, 24 h, in air	[CL]:[Cat] = 500:1	26	3820	1.44
MOF-74-Mn[b]	130 °C, 24 h, in air	[CL]:[Cat] = 500:1	96	2290	2.58
**2** (This paper)	130 °C, 24 h, in air	[VL]:[Cat] = 500:1	99	12670	2.29
MOF-74-Mn[b]	130 °C, 24 h, in air	[VL]:[Cat] = 500:1	96	3470	2.07
**3** (This paper)	130 °C, 24 h, inert atmosphere	[CL]:[Cat] = 500:1	22	2910	1.14
Zn-DABCO[9]	160 °C, 4 h, inert atmosphere	[CL]:[Cat] = 100:1	-	-	-
Zn-CP-NO_2[10]	160 °C, 24 h, inert atmosphere	[CL]:[Cat] = 1000:1	44	23100	1.05
Zn-CP-H[10]	160 °C, 72 h, inert atmosphere	[CL]:[Cat] = 1000:1	8	49800	1.49
MOF-74-Zn[10]	160 °C, 72 h, inert atmosphere	[CL]:[Cat] = 1000:1	13	15600	1.22
**3** (This paper)	130 °C, 24 h, inert atmosphere	[VL]:[Cat] = 500:1	61	9190	1.25
ZIF-8[7]	140 °C, 9 h, inert atmosphere	[VL]:[Cat] = 100:1	93	11800	1.45

[a] The mol ratio of [Monomer]:[Catalyst]. [b] Synthesized following the literature method[11].

References:

1 R. W. W. Hooft, COLLECT data collection software, Nonius B.V., (1998).

2 Z. Otwinowski and W. Minor, Processing of X-ray diffraction data collected in oscillation mode, Methods in Enzymology, *Macromolecular Crystallography*, Pt. A (1997), **276**, 307-326, C. W. Carter, Jr. & R. M. Sweet, Eds., Academic Press.

3 *CrysAlis PRO* 1.171.43.98a software (Rigaku Oxford Diffraction, 2023).

4 Sheldrick, G. M., (2008), *Acta Crystallogr.* A64, 112-122. A short history of SHELX.

5 G. M. Sheldrick, *Acta Cryst*. (2015), **A71,** 3-8. SHELXT – Integrated space-group and crystal structure determination.

6 G. M. Sheldrick, *Acta Cryst*. (2015), **A71,** 3-8. SHELXT – Integrated space-group and crystal structure determination.

7 F. Naz, B. Mousavi, Z. Luo, C. Jabbour, P. M. Heynderickx, S. Chaemchuen and F. Verpoort. *Appl Organometal Chem*., 2019, **33**, e4890.

8 N. Shen, F. Tian, J. Chang, K-L. Huang, Z-H. Zhang, X. Feng, J. Gu, S-C Chen, M-Y. He and Q. Chen. *CrystEngComm*, 2020, **22**, 3656.

9 F. Naz, M. Ciprian, B. Mousavi, S. Chaemchuen, M. Zhu, S. Yan and F. Verpoort. *European Polymer Journal*, 2021, **142**, 110127.

10 Z. Fang, Y-J. Z, D. Wang, Z. Wang, C. Lian, K-L. Huang, J-F Qian, S-C Chen and Q. Chen. *Molecular Catalysis*, 2024, **569**, 114507.

11 S. Xie, Q. Qin, H. Liu, L. Jin. X. Wei, J. Liu, X. Liu, Y. Yao, L. Dong and B. Li. *Appl. Mater. Interfaces*, 2020, **12**, **43**, 48476-48485.
